# Stimuli-responsive hydrogels: smart state of-the-art platforms for cardiac tissue engineering

**DOI:** 10.3389/fbioe.2023.1174075

**Published:** 2023-06-28

**Authors:** Hussein M. El-Husseiny, Eman A. Mady, Walaa A. El-Dakroury, Ahmed S. Doghish, Ryou Tanaka

**Affiliations:** ^1^ Laboratory of Veterinary Surgery, Department of Veterinary Medicine, Faculty of Agriculture, Tokyo University of Agriculture and Technology, Fuchu, Japan; ^2^ Department of Surgery, Anesthesiology, and Radiology, Faculty of Veterinary Medicine, Benha University, Benha, Egypt; ^3^ Laboratory of Veterinary Physiology, Department of Veterinary Medicine, Faculty of Agriculture, Tokyo University of Agriculture and Technology, Fuchu, Japan; ^4^ Department of Animal Hygiene, Behavior and Management, Faculty of Veterinary Medicine, Benha University, Benha, Egypt; ^5^ Department of Pharmaceutics and Industrial Pharmacy, Faculty of Pharmacy, Badr University in Cairo (BUC), Badr, Egypt; ^6^ Department of Biochemistry, Faculty of Pharmacy, Badr University in Cairo (BUC), Badr, Egypt; ^7^ Biochemistry and Molecular Biology Department, Faculty of Pharmacy (Boys), Al-Azhar University, Cairo, Egypt

**Keywords:** stimuli-responsive hydrogels, cardiac tissue engineering, tissue engineering, drug delivery, myocardial infarction

## Abstract

Biomedicine and tissue regeneration have made significant advancements recently, positively affecting the whole healthcare spectrum. This opened the way for them to develop their applications for revitalizing damaged tissues. Thus, their functionality will be restored. Cardiac tissue engineering (CTE) using curative procedures that combine biomolecules, biomimetic scaffolds, and cells plays a critical part in this path. Stimuli-responsive hydrogels (SRHs) are excellent three-dimensional (3D) biomaterials for tissue engineering (TE) and various biomedical applications. They can mimic the intrinsic tissues’ physicochemical, mechanical, and biological characteristics in a variety of ways. They also provide for 3D setup, adequate aqueous conditions, and the mechanical consistency required for cell development. Furthermore, they function as competent delivery platforms for various biomolecules. Many natural and synthetic polymers were used to fabricate these intelligent platforms with innovative enhanced features and specialized capabilities that are appropriate for CTE applications. In the present review, different strategies employed for CTE were outlined. The light was shed on the limitations of the use of conventional hydrogels in CTE. Moreover, diverse types of SRHs, their characteristics, assembly and exploitation for CTE were discussed. To summarize, recent development in the construction of SRHs increases their potential to operate as intelligent, sophisticated systems in the reconstruction of degenerated cardiac tissues.

## Graphical Abstract

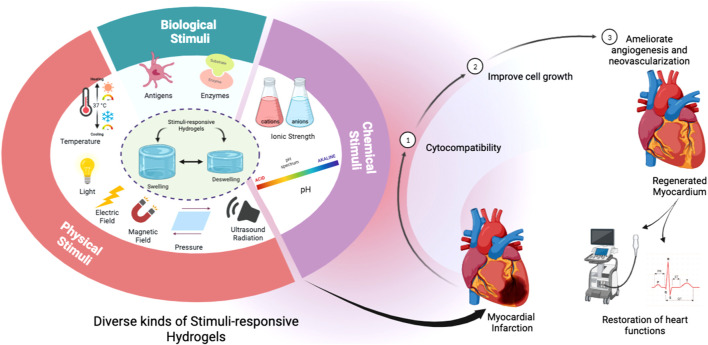

## 1 Introduction

**FIGURE 1:**
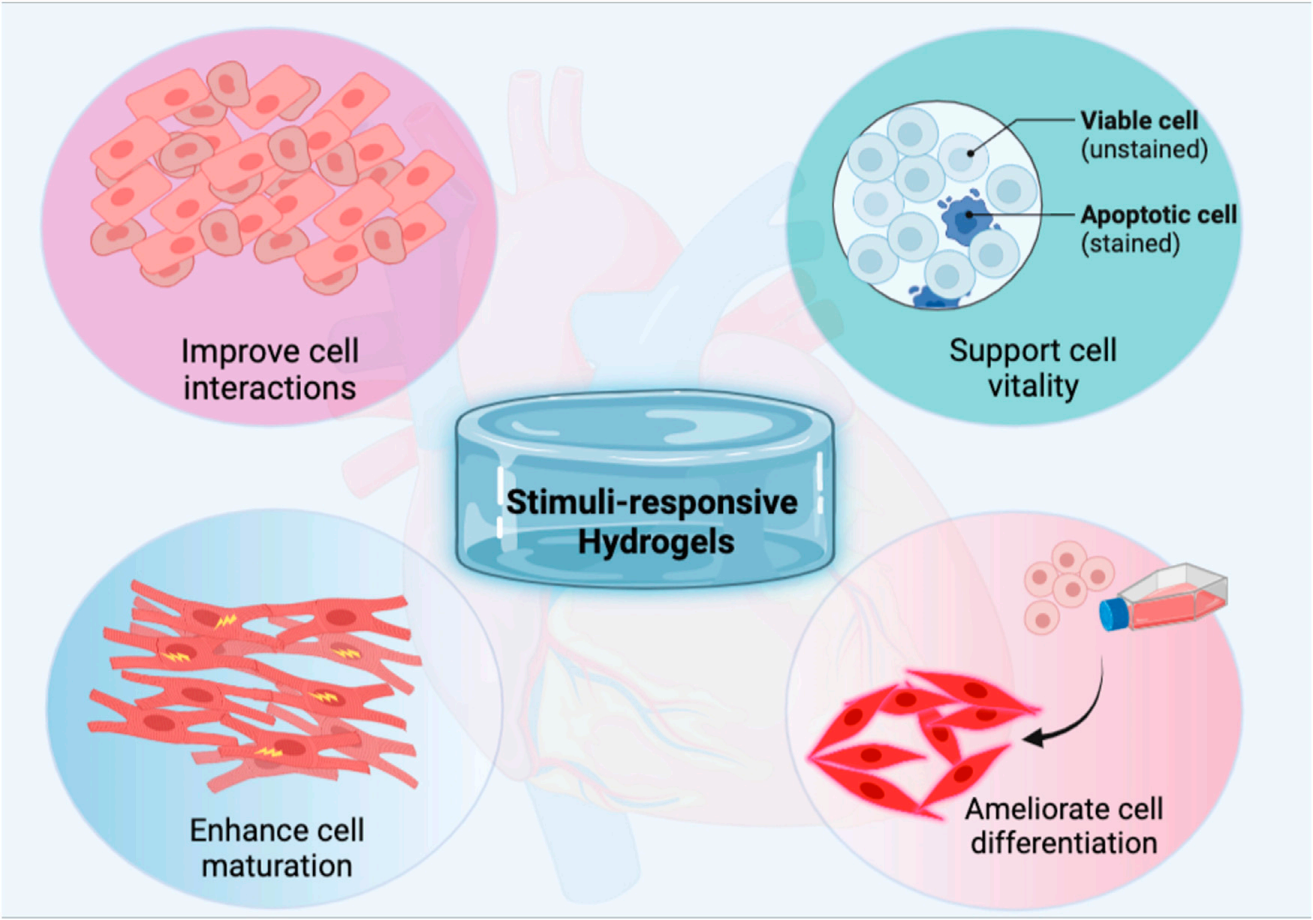
Schematic illustration of the pivotal role of stimuli-responsive hydrogels to ameliorate cardiac tissue engineering.

Cardiovascular diseases (CVDs) are the leading cause of mortality worldwide, with the number of individuals dying from CVDs growing every year (WHO, 2020). Many CVDs due to diverse causes may now be detected and even predicted early because of advancements in heart function measurement tools (Mandour et al., 2020; Ma et al., 2021; Yairo et al., 2021; Yoshida et al., 2021; El-Husseiny et al., 2022a; El-Husseiny et al., 2022e; Ma et al., 2022; Sasaki et al., 2022; Yoshida et al., 2022; Abulsoud et al., 2023; El-Husseiny et al., 2023b). Given the restricted ability of human cardiac cells to regenerate completely, several tissue engineering (TE) techniques are used to circumvent this obstacle. To heal cardiac and vascular tissues, a variety of materials have been used (Kiritani et al., 2020; Tanaka et al., 2020; House et al., 2021). Furthermore, a variety of hydrogels have been employed to overcome this constraint. The basic goal of TE is to repair damaged tissues and replace them with new biological ones (Gaspar et al., 2020; Lavrador et al., 2021; El-Husseiny et al., 2022c; El-Husseiny et al., 2022d; El-Husseiny et al., 2022f; Sharun et al., 2023). Cell biology and biochemistry are required for this interdisciplinary process (El-Husseiny et al., 2022d; Doghish et al., 2023; El-Mahdy et al., 2023; Mady et al., 2023; Sharun et al., 2023). For clinical applications, clinical medical and material sciences investigations are also included (Gaspar et al., 2020). The physiologically active platforms are porous, 3D structures that allow biologically active components such as biomolecules, proteins, and growth factors to be attached to their surface. These biosystems’ ability to contribute particular bioactivity to scaffold construction verifies its unique promise in TE and other biomedical applications (Mantha et al., 2019). They can be used to deliver medicines and bioactive peptides, as well as filling agents and 3D structures. They can also regulate the regeneration process and encourage the creation of the needed tissue (Khandan et al., 2017). Here, the biomaterial must have the right characteristics to fit the needs of tissue regeneration. Cell seeded bioactive materials serve an important part in the development of newly formed tissue by directing self-seeded cell growth or encouraging cell migration. They also serve as cell delivery matrices to certain bodily tissues. Furthermore, they are involved in the integrity of freshly formed tissue structure and function (Zhu and Marchant, 2011). They must have the essential physico-chemical characteristics that cause cell adhesion to their surfaces, cell growth, multiplication, differentiation, and migration for these objectives (Silva et al., 2017) as presented in Figure 1, and to avoid the adverse consequences of a lack of these qualities, such as cell necrosis and impaired tissue regeneration (Silva et al., 2017; Mantha et al., 2019).

Several biological platforms have been developed over the years from natural sources such as algae (Silva et al., 2017), and animal tissues (El-Husseiny, 2017; El-Husseiny et al., 2019), as well as synthetic sources such as lactic acid (Narayanan et al., 2016), and glycoside monomers (Donaire et al., 2009), and caprolactone (Ahmed, 2015; Liu et al., 2018). Even though many polymeric scaffolds utilized for CTE might provide critical support and assets, they lack several critical qualities such as appropriate cell mimicking and acceptable contact with stromal cells. Among them were collagen-fibrin-based hydrogels implanted with cardiomyocytes (CMs) produced from human-induced pluripotent stem cells (hiPSCs-CM) to restore the damaged cardiac tissue (Kaiser et al., 2018). This combination of stem cell-laden hydrogel scaffolds has shown promise and played a key role in CTE. Another micro-channeled 3D printed gelatin (Gt)-based hydrogel system was created to stimulate cardiac cell proliferation while also allowing stem cells to be used to improve heart regeneration (Tijore et al., 2018). However, given the rapid advancement of hydrogel-based systems for heart regeneration, more research is needed to overcome the current constraints. Hydrogel-based scaffolds might provide a suitable, less invasive alternative that could support the qualities that conventional scaffolds lack (Mantha et al., 2019). Hydrogels are water-based hydrophilic 3D polymeric structures. They might be natural, synthetic, or semi-synthetic. Extracellular matrix (ECM), epidermis, mucous, cartilage, meniscus, Gt, collagen, tendons, and vitreous humor are some of the bodily structures that include them (Ahmed, 2015; Liu et al., 2018). They were proposed as innovative materials with promising results in TE. They may transport a large amount of water or biological fluids due to their hydrophilic nature, in which nutrients dissolve and diffuse to cells. Within primarily aquatic settings, their cross-linking structure maintains their integrity and avoids dissolution. Furthermore, they provide support to neighboring cells by adopting a high level of elasticity and flexibility, similar to the original ECM (Maitra and Shukla, 2014). The perfect bioscaffold can bridge tissue defects and improve repair by stimulating new tissue development and neovascularization while also demonstrating high levels of incorporation and biodegradation since they should vanish during or shortly after healing (O’brien, 2011). Hydrogels are the preferred scaffold for a variety of biomedical and TE applications because of their unique structure and properties (Fan et al., 2019b). However, increasing their therapeutic use by making them more adaptable to ongoing body functioning and pathological changes remains a challenge. Thus, new hydrogel materials with smart characteristics capable of promoting clinical applications in biomedicine and TE are still needed. Researchers are increasingly recognizing that recapitulating original tissues’ intrinsic reactive capacity to biophysical and biochemical cues is a key component for improving functional tissue restoration. This rapidly expanding notion is leading to the development of smart hydrogel platforms that can respond to stimuli on demand (Lavrador et al., 2018; Badeau and DeForest, 2019). When realizing and responding to either internal physiological or external applied stimuli, such smart designs provide unprecedented monitoring over network assembly/disassembly, selective biomolecule presentation, and other customizable qualities. (Lavrador et al., 2021). The development of hydrogel polymeric scaffolds in various biomedical applications will be discussed in this review, with a focus on TE. Aside from current advancements in stimuli-responsive hydrogels (SRHs) and their future perspectives in regenerative medicine.

## 2 Physico-chemical, mechanical, and biological properties of the cardiac tissue

The endocardium, myocardium, and epicardium are the three layers of the heart wall. Cellular and extracellular matrix (ECM) composition within these layers varies to optimize cardiac tissue function (Hendrickson et al., 2021). On the luminal side of the heart, endothelium lines the chambers, and on the other side, smooth muscle fibers are tangled with connective tissue to form the endocardium. Myocardial cells communicate with the endocardial cells below them via the subendocardial layer. This connects to the conduction system of the heart, where signal-conducting cells work together (Zhou and Pu, 2016). Although cardiomyocytes (CMs) only make up around a third of the myocardial cells, their volume represents a whopping 75 percent of the heart’s total volume. Specialized conduction cells, endothelial cells, fibroblasts, and immune cells are only a few of the additional cell types found in the heart (Perbellini et al., 2018).

Fibrous proteins (collagen, elastin), sticky glycoproteins (laminin, fibronectin), and proteoglycans make up a relatively modest portion of the extracellular matrix (ECM) of the heart, but they play a crucial role in establishing the mechanical characteristics and compliance of cardiac tissue (Parker and Ingber, 2007). Together, these parts build a sophisticated three-dimensional structure that helps cells maintain their shape as they shrink. The heart wall is distinguished by the presence of interlaced structural proteins like collagen and elastin, which help to force transmission during contraction and prevent the wall from overstretching in various directions (Parker and Ingber, 2007). Each CM is enveloped by a basement membrane made of collagen IV and V, which links it to the remainder of the collagen network and the elastin bundles. Endomysium, perimysium, and epimysium are the three distinct network types that make up this collagen and elastin network’s complex, hierarchical design. Each CM is surrounded by endomysium, which facilitates communication between cells. It is believed to serve a crucial function in establishing links between the contractile apparatuses of neighboring CMs. Most of the endomysium is made up of collagen III, which gives matrix compliance, collagen I give it rigidity and toughness, and elastin gives it elasticity (Yang et al., 2015). The perimysium wraps itself tightly around groups of muscle fibers to strengthen the heart’s wall muscles (Yang et al., 2015). The epimysium wraps around several bundles, and its alignment with the CMs’ axis of symmetry during the cardiac cycle aids in preventing the CMs from being overstretched. Because of its complex composition and architecture, the heart may contract with great force and stress on the cells while still preserving tissue integrity and allowing for the survival of the cells (Yang et al., 2015).

Aging and disease can wreak havoc on the cardiovascular system by damaging its intricately ordered architecture and so affecting the way cells in the system communicate and contract with one another (Eghbali and Weber, 1990). An example is cardiac fibrosis, where there is a significant reorientation of cell-cell connections and a severe change in myofibrillar thickness, both of which lead to reentrant arrhythmias and aberrant contractile performance. The heart’s ECM is analogous to a grid that may physically organize CMs, allowing for their coordinated contraction; once the pattern is disrupted for any cause, the conduction system fails (Eghbali and Weber, 1990; Howe et al., 1991).

During embryogenesis, the heart receives a number of mechanical cues that help guide its growth. Around day 20 of embryonic development, the first heartbeat is detected. This feeble heartbeat gradually strengthens in amplitude and frequency as cells undergo remodeling in response to the beating’s mechanical stimulation (Howe et al., 1991). The average heart rate is 80 beats per minute (1.33 Hz) on day 26, increasing to between 160 and 200 beats per minute (2.67 and 3.33 Hz) by day 45. Mechanical stress on the heart is raised when sarcomeric remodeling and cell junctional alignment work together to increase heart rate and contraction strength (Taber, 1998). Through mechanical signals, this promotes even greater sarcomeric remodeling, cell alignment, and extracellular matrix (ECM) remodeling. As a result, the biophysical microenvironment is essential for the proper growth and maintenance of heart tissue. For the heart to handle the increased mechanical stress and strain it experiences throughout the typical cardiac cycle, focal adhesions, integrins, and other mechanosensory on the membrane of CMs serve to distribute the mechanical load (Taber, 1998). Again, extracellular matrix (ECM) components are crucial, not only because they serve as a substrate for cell attachment but also because integrin-mediated mechanotransduction affects ion channel conformational alterations (Zhu et al., 2014). Cytoskeletal proteins govern this connection, which in turn causes ion channel-regulated action potentials and contraction in response to mechanical pressures applied to cells via integrin receptors. However, many indicators suggest that the intricate heart architecture contributes not only to favor the conductivity, as mentioned above but also to the heart’s contract ability, suggesting that the role of specific ECM-dependent molecular pathways in mediating the coordinated contraction of CMs is an understudied field (Frangogiannis, 2019).

The mechanical properties of heart tissue, such as Young modulus, tensile strength, and elongation %, reveal its unique characteristics (Akhmanova et al., 2015). These values can be gleaned through direct mechanical failure testing on tissue samples. The stress-strain curve can be obtained by measuring the applied force and the sample’s elongation (Hendrickson et al., 2021). After a certain point on the stress-strain curve, the sample’s section starts decreasing (perpendicular to the applied force), indicating poor mechanical qualities (Majkut et al., 2013). This point corresponds to the tissue’s tensile strength, which represents its capacity to sustain tension. The tensile strength of the heart is between 1 and 15 kPa. These characteristics can be traced back to fundamental elements of the heart’s ECM. Collagen, as the principal ECM component in modifying tensile strength, specifically contributes to the heart’s outstanding capacity to tolerate the deformation that happens during contraction (Muiznieks and Keeley, 2013). The major function of elastin is to provide elasticity, allowing the tissue to return to its original place when a contraction cycle has ended. Molecular perturbations caused by fibrosis’ dysregulation of ECM deposition lead to the degradation of structural proteins, which in turn disrupts ECM-dependent pathways that govern CMs’ contraction and cause systolic dysfunction (Muiznieks and Keeley, 2013).

Alterations in the conduction and contraction between cells in the cardiovascular system can come from damage to this intricate and well-organized architecture brought on by aging or disease. For instance, cardiac tissue fibrosis, which is primarily a malfunction of the extracellular matrix of the tissue, causes reentrant arrhythmias and aberrant contractile function due to a significant reorientation of cell-cell connections and a severe change in myofibrillar thickness. The heart’s ECM can be thought of as a grid that physically organizes CMs, allowing for their coordinated contraction; once the pattern is disrupted for any cause, the conduction system fails (Muiznieks and Keeley, 2013).

## 3 Current regeneration strategies for cardiac tissue engineering

**FIGURE 2:**
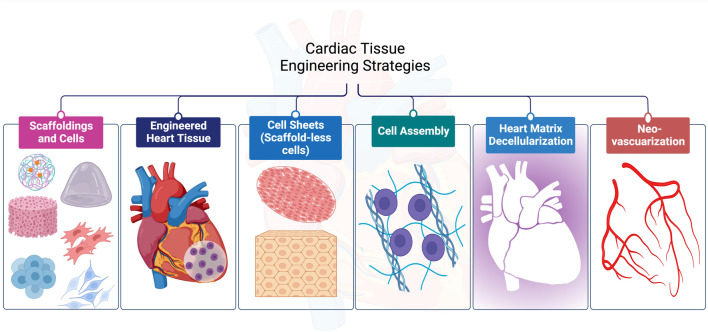
Diverse strategies are employed for cardiac tissue engineering applications.

For CTE, diverse techniques have been employed successfully as shown in Figure 2. In this section, we will discuss them in detail with an emphasis on the materials used and the limitations of each strategy.

### 3.1 Cell based therapy

#### 3.1.1 Scaffoldings and cells

Stem cells (SCs) are multipotent, self-renewing cells that play an essential role in maintaining and repairing tissues (Hendawy et al., 2021; El-Husseiny et al., 2023a). Tissue repair relies heavily on the use of autologous stem cells. Tissues with a high capacity for regeneration, like skin, only need a little insult before the activated stem cells can heal the damage. Nevertheless, adult hearts’ ability to regenerate was restricted, and cardiac SCs (CSCs) were insufficient to replace the damaged CMs. (Cotsarelis, 2006).

Recent advancement has been made in inducing pluripotent SCs (iPSCs) out of adult cells via reprogramming with specified transcription factors (Takahashi et al., 2007; Yu et al., 2007; Shi et al., 2011). The iPSCs have been used to generate a wide variety of cardiovascular cells, and a recent research demonstrated that postnatal cardiac or cutaneous fibroblasts may be directly transformed into cardiomyocyte-like cells (Ieda et al., 2010; Shi et al., 2011). While personalized iPSCs or cardiomyocyte-like cells opened up a new avenue for autologous stem cell therapy, the risk of tumor development has arisen (Rolletschek and Wobus, 2009; Elfadadny et al., 2021). Heart CMs may be derived from many different types of SCs, including mesenchymal SCs (MSCs), embryonic SCs (ESCs), skeletal progenitor cells (SPCs), hematopoietic SCs, and cardiac progenitor cells (CPCs) (Shi et al., 2011; Nagaya et al., 2005; Zhang et al., 2004; Orlic et al., 2001; El-Husseiny et al., 2023a).

Even though exogenous stem cell transplantation therapy has received a lot of attention, most allogeneic cells go through necrosis or apoptosis after transplantation due to immunoreaction and inadequate surroundings. Furthermore, the employment of ESCs therapy has been restricted by ethical issues (Chien, 2008; Shi et al., 2011). Although several investigations have reported that cytokines can mobilize autologous SCs, there is no functional way to selectively attract SCs to cardiac damage sites. Furthermore, SCs need a sequence of coordinated interactions with their biological surroundings, whether they were injected directly or attracted by mobilization factors. One of the main challenges for transplanted SCs is the harsh microenvironment inside injured host cardiac tissue. Low oxygen levels, inflammatory mediators, oxidative stress, and deprived nutrient supply impede successful engraftment all work against engraftment and hasten cell death following a transplant (Karpov et al., 2017).

Heart failure has been treated with tissue-engineered cardiac patches such as ECM, however, the potency of myocardial healing was restricted due to the restricted ability for cell infiltration (LIU et al., 2008; Sapir et al., 2011). To imitate the various interactions between heart cells and the ECM, decellularized platforms from porcine or rat myocardium have recently been used (Bergmeister et al., 2008; Sapir et al., 2011). Although decellularized matrices are found to be favorable when it comes to CTE, their application in transplantation has high risks, including a scarcity of human donors, and other immunological concerns, (Sapir et al., 2011). Hence, researchers are now focusing their efforts on developing ECM biomimetic platforms made of synthetic and/or xeno-free biomaterials (Sapir et al., 2011; Shachar et al., 2011). Biomaterials are being used more and more to target repair (Chen et al., 2007; Han et al., 2009; Chen et al., 2010; El-Husseiny, 2017; El-Husseiny et al., 2019). They could function as a network for cell endurance, propagation, and multiplication, and as a guide for reestablishing 3D tissue (Davis et al., 2005; Shi et al., 2011). Biomaterials are being established as cardiac patches to increase heart function via supporting and repairing damaged regions by replacing damaged myocardial tissues or scar tissue (Dvir et al., 2009; Zhang et al., 2009).

#### 3.1.2 Cell sheet technique (scaffold-less cell)

Although though novel biomaterial features have been developed to address the challenges associated with biomaterial systems, such as integration impairment with host tissues, immunogenicity, and undesirable breakdown products, these problems persist (Feric and Radisic, 2016; Allen, 2018). Scaffold-free engineered tissues known as cell sheets could be created using intact cell monolayers non-enzymatically isolated from substrates *in vitro* as an alternative (Allen, 2018). It is a strategy that has therapeutic application potential. Poly (N-isopropylacrylamide (PNIPAAm) is used for covering a thermosensitive cell culture layer. This polymer is cell adherent at 37°C and changes characteristics in reverse below 32°C. It is also possible to extract cells off a culture dish as a cell sheet once they have aggregated and developed gap junctions by lowering the temperature (Shimizu et al., 2002; Tee et al., 2010). Furthermore, the capability to layer individual CM cell sheets into a 3D contractile cardiac tissue was established *in vivo* (Shimizu et al., 2006). The tissue endured subcutaneous implantation for 1 year, and cell sheets were functionally integrated with the host’s heart when applied to rat hearts (Furuta et al., 2006). Also using an orbital shaker, Stevens and his group have created a scaffold-free CM cell patch (Stevens et al., 2009). In both scenarios, the result is tissue that is identical to a compact myocardium but without the scaffold. Limitations of this method are the contemporary need for certain vascularization strategies and nutrient diffusion supply to maintain the patch’s durability and adaptability for the development of thick myocardial tissue constructs (Tee et al., 2010).

#### 3.1.3 Cell assembly

It is not necessary to plant cells into 3D porous scaffolds. Instead, cells can be suspended in hydrogel-based scaffolds, that provide a suitable environment for them to travel and arrange into contractile tissues whether *in vitro*, by gravity-enforced methods to form sphere-like tissues (Kelm et al., 2004; Kelm et al., 2006; Tee et al., 2010), or *in vivo*, by an arteriovenous loop (AVL) embedded chamber for the vascularization of the arranged CMs (Birla et al., 2005; Morritt et al., 2007; Tee et al., 2010).

The viscoelastic properties of hydrogels and their adaptability to chemical and physical changes have attracted substantial attention as cardiac tissue constructions (Slaughter et al., 2009; Radhakrishnan et al., 2014). They are water-insoluble polymers that can absorb a large quantity of water or biofluids, causing swelling and an expansion of their dimensions while keeping their shape. This feature makes them very close to soft tissues in their structure and function (Wu et al., 2008; Jeong et al., 2012; Radhakrishnan et al., 2014). It is likely to modify the surface of a hydrogel to have it respond to a certain stimulus, such as temperature, pH, molecules, magnetic or electric signals, and ionic strength (Kopeček, 2007).

Since typical hydrogels are quite often formed into larger sizes that have low surface-to-volume ratios, they have slow degradation values and limited cell infiltration along with weak vascularization. Hydrogels of this type have only nanoporous meshes within the cross-linked networks and lack micropores, indicating that nutrient transfer and cell vitality are insufficient within hydrogels (Allazetta and Lutolf, 2015; Hsu et al., 2019; Feng et al., 2021). It was found that replacing bulk hydrogel with microporous annealed particle (MAP) that possesses a larger surface/volume percent and shorter diffusion distance can boost the mass movement of nutrients and promote long-term cells survival. Its pores can help guide cell multiplication and tissue development before the hydrogel breaks down (Ma et al., 2017; Feng et al., 2019; Feng et al., 2020; Feng et al., 2021).

Blood-derived MSCs are essential infiltrating cells that have a predisposition to relocate to the myocardial infarction (MI) region. It was hypothesized that vascular endothelial growth factor (VEGF)-encapsulated MSCs aimed at MI tissue could enhance the cardiac activity via angiogenesis and the MSCs’ tropism to the MI area (Liu et al., 2017a). Angiogenesis and heart function was improved by employing self-assembled alginate (Alg.) and Gt polyelectrolytes in the first stages of development. SDF-1 was found to be an attractive target for the VEGF-encapsulated MSCs *in vitro* with a stable release of VEGF. *In vivo*, angiogenesis was stimulated in the MI region by VEGF-encapsulated MSCs, and cardiac functions were enhanced. For MI treatment, these preclinical data imply that this VEGF-loaded layer-by-layer self-assembled encapsulated MSCs may be an effective and minimally invasive treatment option for MI (Liu et al., 2017a).

When self-assembling peptides are situated in a physiological environment, they create stable nanofiber hydrogels (Segers et al., 2007; Radhakrishnan et al., 2014). As a consequence of the *in situ* injection of RAD16-II peptide gels that self-assembled in the myocardial, it induced an appropriate microenvironment (Davis et al., 2005; Radhakrishnan et al., 2014). Endothelial cells, non-vascular cells, and smooth myocytes were recruited by this microenvironment. RGDSP sequence with a cell-adhesive domain was connected to the self-assembling peptide RAD16-I to produce a biomimetic self-assembling peptide. The produced scaffold enhanced the adherence and viability of marrow-derived CSCs and facilitated their propagation to develop CMs, which as a result, improved heart activity and repair (Guo et al., 2010).

### 3.2 Biomaterials

#### 3.2.1 Engineering of the cardiac tissue

The classic TE process involves seeding target cells into a scaffold *in vitro*, sometimes with modification (e.g., special conditioned culture) (Radisic et al., 2006; Tee et al., 2010), and then implanting the construct *in vivo,* for both preclinical animal studies (Golas et al., 2014), and human clinical applications (Rana et al., 2017). Biomedical alternatives such as biomaterials are constantly being explored in the field of TE for the entire (or partial) replacement of injured tissue. The advance of a 3D matrix as a platform is a marked role for biomimetic materials. The biomaterials must also be suitable for the conservation of the cells and the signals necessary for tissue or organ regeneration. Following that, regenerated tissues need to maintain, reinstate and augment function (Ma, 2008; Balakrishnan and Banerjee, 2011; Gauvin and Khademhosseini, 2011; Iqbal et al., 2018). Whenever cardiac constructs had been implanted in a damaged myocardial area, neovascularization from the epicardium infiltrated the graft, and distributed fetal CMs survived the implantation process (Li et al., 1999; Tee et al., 2010). Upon implantation in the patient, tissue-engineered materials may become functional at the implantation time or be capable of integrating and accomplishing the predicted function following implantation. As in either instance, the biomaterial needs to integrate well with the recipient or transplanted cells to effectively share in the tissue regeneration via cell-cell signaling and the release of growth factors (GFs), propagation, multiplication, and development of ECM (Vlierberghe et al., 2011; Peng et al., 2016; Jakus et al., 2017; Pacelli et al., 2017; Ravnic et al., 2017; Wu et al., 2017; Iqbal et al., 2018).

Intrinsic tissue regeneration for the heart is not a portion of existing therapy for multifaceted cardiovascular injury (Bergmann et al., 2009; Iqbal et al., 2018). Relationships between tissue regeneration, engineered biomaterials, and our immune system have to be entirely understood. The objective of cellular and TE is the evolvement of treatments that will stabilize, alter, or improve cardiovascular physiology and anatomy. Polymeric systems used in CTE have been outlined and constructed using different approaches (Puppi et al., 2017; Iqbal et al., 2018). They might be employed in the fabrication of degradable cardiac patches for example. In the long term, these polymeric biodegradable cardiac patches can provide excellent circumstances for cellular growth (Silvestri et al., 2013). Studies on elastomeric biodegradable poly (glycerol sebacate) (PGS), like Gt Nano-fibrous scaffolds, and PGS/fibrinogen core/shell fibers, have been conducted. Anisotropy was established in these materials, imitating the left ventricular (LV) myocardium. This can be employed as a construct for myocardial regeneration (Kharaziha et al., 2013; Ravichandran et al., 2013). The cells’ cytoskeletal organization was influenced by the scaffolds’ structural features. For example, the amalgamation of synthetic Poly (lactic-co-glycolic acid) (PLGA) with natural Gt polymers was produced via electro-spinning by Prabhakaran et al. to create PLGA/Gel Nano-fibers. The potential of these scaffolds as biomimetic cardiac patches was highlighted by culturing the cardiomyocyte cells on them (Prabhakaran et al., 2011; Iqbal et al., 2018).

Engineered heart tissue (EHT) is a spontaneously contractile construct created using neonatal rat CMs, collagen I matrix, Matrigel™, and a mechanical stretching device. It is amongst the most promising CTE approaches (Zimmermann et al., 2000; Zimmermann et al., 2002a; Zimmermann et al., 2006; Yildirim et al., 2007; Tee et al., 2010). It was originally designed as a planar structure (Zimmermann et al., 2000), which was then transformed into a circular structure (Zimmermann et al., 2002b) that had greater contractile qualities and a more distinct CM structure. For *in vivo* testing, the construct’s 3D geometry was further changed into a pouch-like structure (Yildirim et al., 2007), which was then used to “encase” a failing heart to function as a biological ventricular assist system. There have been improvements to the *in vitro* states to lessen the utilization of serum and Matrigel™ as well. Although the model looks to exhibit significant potential, core tissue viability is minimal, and a more advanced version of the EHT is still needed (Tee et al., 2010).

#### 3.2.2 Heart matrix decellularization

When cells are removed from organs or tissues while the ECM is left intact, it is called decellularization (Rodrigues et al., 2018). Decellularized ECM has been established based on the idea that native ECM might be a better substitute for a tissue’s complex environment (Radhakrishnan et al., 2014). This can be achieved in diverse ways: chemically, physically, and enzymatically (El-Husseiny, 2017; Rodrigues et al., 2018; El-Husseiny et al., 2019). Although this could alter the ECM’s biochemical and morphological components, it has the advantage of removing cellular antigens which can trigger a foreign body reaction via inflammation, antibody activation, and probable transplant rejection (Radhakrishnan et al., 2014). Biologic scaffolds used in clinical applications are produced using this technique. However, it has been proven that using perfusion decellularization preserves the organs’ 3D geometry while removing the cells in a more uniform manner (Tapias and Ott, 2014; Keane et al., 2015; Rodrigues et al., 2018). To develop an entire-heart ECM scaffold, rats’ hearts were decellularized. The scaffold maintained its 3D structure, with the vascular tubing skeleton preserved by the presence of vascular basement membranes (Ott et al., 2008; Tee et al., 2010). A spontaneous contractile complete heart was generated after planting neonatal rat EC and CMs under physiological circumstances. This method has the potential of creating a big human or animal heart to replace the human heart functionally.

**FIGURE 3:**
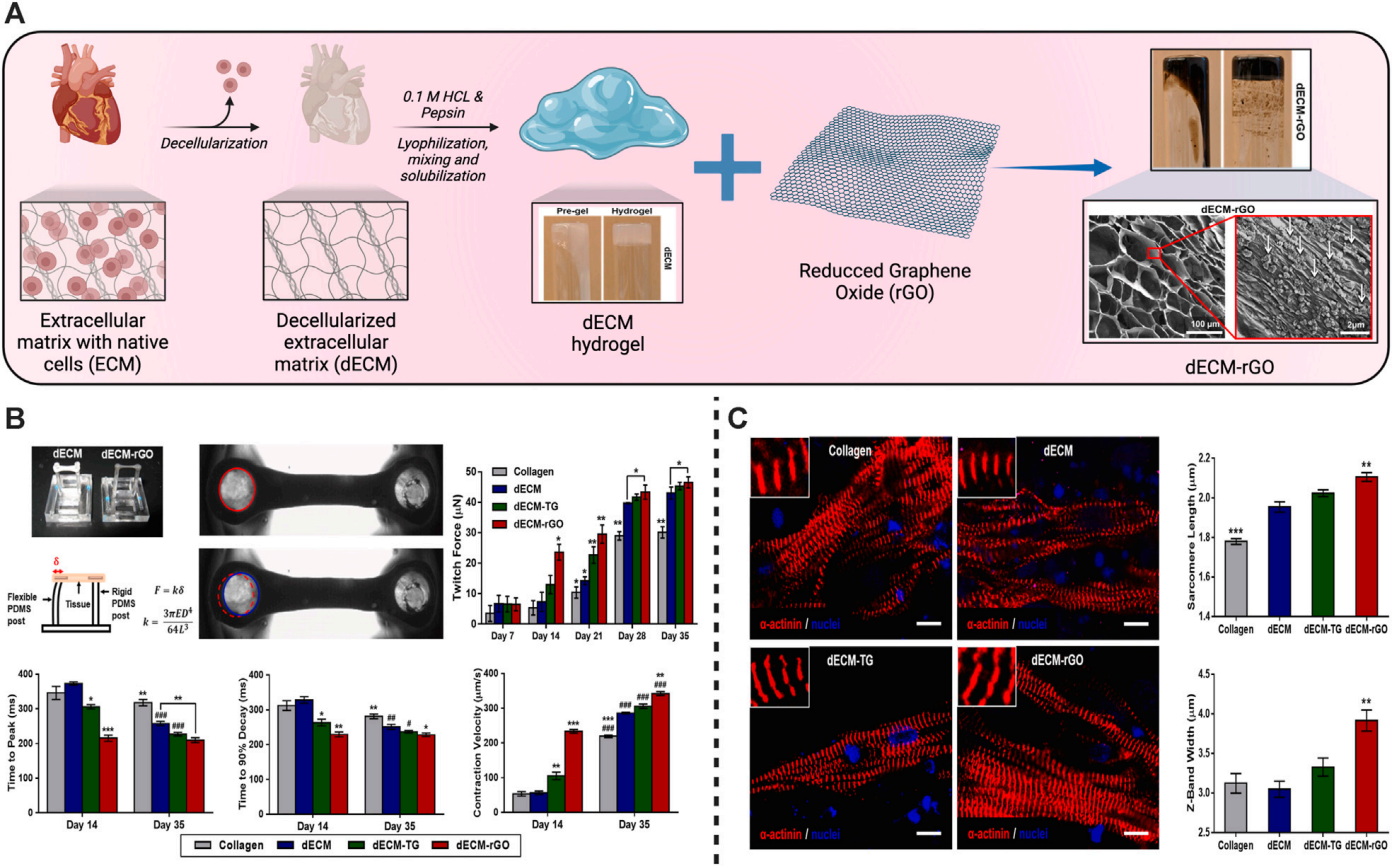
Schematic illustration of the fabrication of hydrogels from the cardiac decellularized extracellular matrix (dECM) for cardiac tissue engineering. **(A)** Schematic of the producers to produce the dECM hydrogels followed by their mixing with reduced graphene oxide (rGO) to synthesize the dECM-rGO composite hydrogels with their structure examined using a scanning electron microscope (SEM). **(B)** Enhancement of the contractile functions and improvement of the twitch forces, maximal twitch forces, relaxation, and contraction velocity of dECM-rGO cardiac tissues. **(C)** Improvement of the sarcomere development in the regenerated cardiac tissues especially in the dECM-rGO hydrogels presented in the fluorescent images with amelioration of the engineered sarcomere length and Z-band width. Copyright © 2021, ELSEVIER Publishing Group. Replicated with permission from (Tsui et al., 2021).

Hydrogels made from the decellularized extracellular matrix (dECM) have gained a lot of interest in recent years due to significant advances in hydrogel technology and theory, as well as advances in the use of dECM hydrogels as a novel regenerative and TE medicine approach. There are structural and stimulatory features of hydrogel responsiveness that are retained along with ECM functionality. dECM hydrogel preserves cell GFs such as transforming GF, Fibroblast GF, and hepatocyte GF, which can improve the seed cell’s proliferation, migration, differentiation, and angiogenesis (Zhang et al., 2021b). They offer the following benefits. 1) The capability to inject. At physiological temperatures, viscous fluids can be polymerized to create hydrogels that adjust to the form of the defective location. 2) The bioactivity of the native matrix is found in dECM hydrogels (Wolf et al., 2012; Zhang et al., 2021b). 3) There is no immunogenic cellular content in dECM hydrogels. 4) The mechanical assets can be altered. 5) Crosslinking or modifying the hydrogel concentration can be utilized to modify the mechanical strength of the dECM (Ungerleider et al., 2015). 6) A gelled dECM has a 3D conformation that is beneficial to cell growth. 7) dECM hydrogels are machinability friendly. It is likely to customize 3D geometric shapes with 3D printing (Lin et al., 2018). Human iPSCs-derived CMs have a significant potency for disease categorizing and drug monitoring when they are exploited to construct human tissues. Hybrid hydrogels were produced by porcine cardiac dECM and reduced graphene oxide (rGO) to support the normal development of cells and tissues (Tsui et al., 2021) (Figure 3). EHTs developed using hiPSC-derived CMs and dECM-rGO hydrogels showed a significant increase in the tension powers and the expression of genes that control contractile function. It also improved many electrophysiological functions, including calcium handling, conductance speed, and action potential period (Tsui et al., 2021).

#### 3.2.3 Neo-vascularization

Most novel blood vessel development occurs in mature organisms because of angiogenesis. Angiogenesis is described as the sprouting and formation of novel microcirculatory vessels from pre-existing vessels (Carmeliet, 2005; Tee et al., 2010). This happens through the breakdown of basement membranes and EC proliferation, which is stimulated by a large variety of growth agents (Page-McCaw et al., 2007). There are only 100–200 mm layers of 6–12 cm thick that can survive in an *in vitro* designed construct that is implanted with CMs and relies only on diffusion for oxygenation and nutrition. Infarcted myocardium’s epicardial surface will be significantly more difficult to neo-vascularize (Suzuki et al., 2009; Tee et al., 2010). There have been diverse ways of vascularizing a clinically significant-sized construct. In general, these procedures can be divided into two categories: *in vitro* and *in vivo* vascularization approaches (Lokmic and Mitchell, 2008; Lovett et al., 2009; Tee et al., 2010).

##### 3.2.3.1 *In vitro* neo-vascularization

In bioengineered tissue constructs, this is described as the growth and manipulation of biological components to generate microvasculature outside of a patient’s own body. This technique is extensively employed in classical TE (Lokmic and Mitchell, 2008; Tee et al., 2010). A CM-seeded cardiac construct is similar to an avascular transplant. For the transplant to survive, it must be infiltrated by the host’s blood vessels. Consequently, the core of the ‘transplant’ will go through necrosis owing to the time it requires for the host vessels to vascularize it. By creating the intrinsic microvasculature *in vitro*, it is conceivable to rejoin the steward vasculature by either inosculation or by surgically connecting the graft to host vessels (Tremblay et al., 2005; Tee et al., 2010).

##### 3.2.3.2 *In vivo* neo-vascularization

*In vivo* neo-vascularization can be further divided into extrinsic/external and intrinsic/internal vascularization: Extrinsic vascularization is the utilization of the patient’s ectopic vasculature as subcutaneous fat, omentum, peritoneum, and axial vessels having the high-angiogenic potential to generate functional microvessels in a non-vascularized engineered construct (Lokmic and Mitchell, 2008; Tee et al., 2010). Omentum was successful in CTE, while peritoneum failed in this regard (Amir et al., 2009). As a clinically significant vascularized graft still requires 3–4 days of “taking”, it relies on diffusion for survival. Therefore, the perfusion timing of these transplants should be carefully considered. If the ECT’s size is large, this may be insufficient to support it (Tee et al., 2010). Regarding intrinsic vascularization, a central macrovascular conduit is put in a protected region to vascularize an endogenously produced or transplanted scaffold. It is employed to develop a microcirculatory network. During reconstructive surgery, the principle of prefabrication of flaps prompted the development of intrinsic vascularization in CTEs (Erol and Sira, 1980; Morrison et al., 1990). When an arterial-vein pedicle was implanted within or under the tissue graft, the tissue would become vascularized and generate an entirely new flap that is based on the pedicle. According to research, the AVL design is the most angiogenic of all pedicle implant configurations (Tanaka et al., 2003; Tee et al., 2010).

## 4 Hydrogel polymeric scaffolds

### 4.1 Classifications of hydrogels

Hydrogels are usually prepared from many natural, artificial (synthetic) polymers, or a hybrid mixture of both types. Alginate (Alg.), chitosan (CH), hyaluronic acid (HA), collagen (Col), and Gt represent the frequently used natural polymers. While poly (acrylic acid) (PAA), poly (acrylamide) (PAAm), polyethylene glycol (PEG), and poly (2-hydroxyethyl methacrylate) are among the frequently used synthetic polymers comprise (Khandan et al., 2017). Hydrogels could be categorized depending on various principles including their polymeric configuration (homopolymeric (Iizawa et al., 2007), copolymeric (Yang et al., 2002), multipolymeric (Maolin et al., 2000; Hacker and Mikos, 2011)), their physico-chemical arrangement (amorphous, semicrystalline, and/or crystalline), sort of crosslinking (chemically, physically, or hybrid crosslinked) (Hacker and Mikos, 2011), their physical appearance (film, matrix, and/or microsphere), and the electric charging of their platform (ionic, non-ionic, zwitterionic, and/or amphoteric electrolyte) (Ahmed, 2015).

### 4.2 Limitations to using conventional hydrogels in cardiac tissue engineering

For years, hydrogels were involved as appealing platforms in many TE applications (Lee and Mooney, 2001). However, the utility of conventional hydrogels for this purpose is sometimes still encountered as a prominent challenge. For instance, several pristine hydrogels lack adequate mechanical power (Burczak et al., 1996). While stable, mechanically robust hydrogels are generally favored for long-term, tension-bearing implementations (Jalili et al., 2016). They display spatial inhomogeneity where the distribution of their crosslinking density is not homogenous which lessens the potency of their networks. Also, since they are fragile, their loading ability and manipulation are problematic. They may need support with further dressing materials to improve their weak adherence capacity. Furthermore, their reduced absorption power renders their therapeutic role in exudative wounds not efficient (Powers et al., 2013). Synthetic hydrogel polymers present limited biocompatibility, and biodegradability that restricted their employment in cardiac tissue engineering (CTE). Difficult sterilization of conventional hydrogels is an additional restriction to use them as they are sensitive to the traditional sterilization techniques accounted to their hydrophilic characters. The strategy of crosslinking affects hydrogels’ release aptitude of biomolecules. Additionally, the chemical cross-linking agents present another threat of toxicity (Li et al., 2009). The previously mentioned challenges have directed the research toward fine-tuning the hydrogels’ characters and supporting their situation as functional systems for applications in CTE. In that context, electrical conductivity is a novel trait that has been recently acquainted with hydrogels to expand their pertinence and additionally the acknowledgment of innovative aptitudes while keeping up their original ones (e.g., tenderness and hydrophilicity) (Chung et al., 2012; Dong et al., 2015; Koutsopoulos, 2016; Khandan et al., 2017; Silva et al., 2017).

## 5 Stimuli-responsive hydrogels for cardiac tissue engineering

The core objective of TE is to renew the degenerated tissues with the restoration of their functions (Khandan et al., 2017). SRHs could express unique characters that make them commonly utilized in TE applications. They serve as platforms to bridge cell migration to the seat of injury, possess an outstanding capacity to provide conditions that mimic the ECM surrounding environment, and could effectively modulate their mechano-physical properties to fit the required application as a tissue defects repair scaffolds (Kopeček, 2007). SRHs have been consumed for the reconstruction of different body tissues, including cardiac tissue (Li et al., 2014; Chen et al., 2017a; Li et al., 2021), neural tissue (Xu et al., 2018; Dong et al., 2020), skin (Yang and Lin, 2004; Rasool et al., 2019; Palem et al., 2021), the cornea (Chiu et al., 2009; Chen et al., 2012; Lin et al., 2017; Hamcerencu et al., 2020), bone (Bush et al., 2016; Zang et al., 2019; Levingstone et al., 2021; El-Husseiny et al., 2022b), cartilage (Zhou et al., 2017a; Mellati et al., 2017; Liang et al., 2018), tendon (Chou et al., 2017; Silva et al., 2018), meniscus (Chen et al., 2020a), and intervertebral disc (Feng et al., 2018; Zheng et al., 2019). Scientists have struggled to design SRHs by amending their physico-chemical features. These smart hydrogels can respond to various physical (temperature, light, electromagnetic fields, pressure, and ultrasound (US) radiation), chemical (pH, glucose, and ionic strength), or biological (enzymes and antigens/antibodies) stimuli and are described as SRHs (Khang, 2017; Lavrador et al., 2021). Different SRHs exploited for CTE are presented in Table 1.

**TABLE 1:** Different stimuli-responsive hydrogels employed for cardiac tissue engineering.

Type of stimuli-responsive hydrogels		Stimuli-responsive hydrogel system	Study model	Purpose of use	Outcomes	References
Physical stimuli-responsive hydrogels	Temperature-responsive hydrogels	Chitosan (CH)-gold nanoparticles GNP loaded with mesenchymal stem cells (MSCs) (CH-GNP/MSCs)	*In vitro* assessment	Cardiac tissue engineering	The integration of GNP could boost the properties of thermo-sensitive CH hydrogels for CTE	Baei et al. (2016)
		Poly (N-isopropylacrylamide (PNIPAAm) containing poly-lactic-co-glycolic acid (PLGA)-encapsulated PVP/H_2_O_2_ core/shell microspheres	*In vitro* (cardiac fibroblast, cardiomyocyte, and endothelial cell)	Treatment of myocardial infarction	Improved survival of the cardiac cells under low oxygen states that imitates the myocardial infarction models	Fan et al. (2018)
		Gellan gum/reduced graphene oxide hydrogel	*In vitro* (rat myoblasts (H9C2))	Development of myocardial tissue engineering scaffold	Gellan gum/reduced graphene oxide hydrogel are promising scaffolds for CTE	Zargar et al. (2019)
		Chitosan (CH)/dextran (DEX)/β–glycerophosphate (β-GP) loaded with umbilical cord mesenchymal stem cells (UCMSCs)	*In vitro* (3T3 cells and human umbilical vein endothelial cells (HUVECs))	Cell delivery carrier for therapy of myocardial infarction	Chitosan (CH)/dextran (DEX)/β–glycerophosphate (β-GP) loaded UCMS is a potential vehicle to deliver cells for CTE	Ke et al. (2020)
		β-glycerophosphate (β -GP) and different kinds of hydrolyzed collagen (HC)-chitosan (CH) hydrogel	*In vitro* (Fetal human ventricular cardiomyocytes cell line RL-14)	Regeneration of the cardiac tissue	The hydrogels found to be promising in increasing cell survival for engineering of infarcted heart tissue	Orozco-Marín et al. (2021)
	Light/Photo-responsive hydrogels	Collagen-polydopamine hydrogel (Col-PDA)	*In vitro* assessment	Control of the cardiomyocyte and neuron activity	Collagen-polydopamine hydrogel (Col-PDA) are promising photo sensitive platforms for applications in tissue engineering	Gholami Derami et al. (2021)
		Cell-degradable poly (2-alkyl-2-oxazoline) (Pox) hydrogel	*In vivo* (rat myocardial infarction model)	Epicardial placement of mesenchymal stem cells for myocardial repair	The synthetic hydrogels are substantial platforms for epicardial delivery of the loaded cells employed for CTE	You et al. (2021)
		Carbon nanotube (CNT)-incorporated photo-cross-linkable gelatin methacrylate (GelMA) hydrogels	*In vitro* assessment	Cardiac engineering and bio actuators	CNT-GelMA are unique multifunctional scaffolds for engineering of infarcted hearts	Shin et al. (2013)
		Gelatin methacrylate-reduced graphene oxide (GelMA-rGO) nanocomposite hydrogels	*In vitro* (cardiomyocytes cell culture)	Cardiac Tissue Engineering	GelMA-rGO hydrogels are outstanding scaffolds for CTE applications *in vitro*	Shin et al. (2016)
	Electro-responsive hydrogels	Poly-3-amino-4-methoxy benzoic acid (PAMB) crosslinked Gelatin (Gt) hydrogels	*In vivo* (rat MI model)	Propagation of the electrical impulse at the MI site to prevent cardiac arrhythmia and preserve ventricular function	Improved ventricular functions and decreased arrhythmia due to the MI in the PAMB-Gt hydrogels treated animals	Zhang et al. (2020a)
		Polyacrylic acid (PAA) mixed with Oxidized alginate (OAlg.)/Gelatin (Gt) hydrogel	*In vivo* (rat MI model)	MI repair	The PAA mixed with OAlg./Gel hydrogel could efficiently reduce cardiac remodeling and improved cardiac function restoration	Song et al. (2021a)
	Magnetic-responsive hydrogels	Chitosan-carbon nanotubes (CH/CNTs) nano scaffold hydrogel	*In vivo* (neonatal rat heart cells)	Cardiac tissue engineering	the integration of carbon nanofibers into the CH platforms improved the characters of scaffolds employed for CTE	Martins et al. (2014)
		Collagen (Col)/magnetic iron oxide (Fe_3_O_4_) nanoparticles coated with Polyethylene glycol (PEG)	*In vitro* assessment	Cardiac tissue engineering	Improved conductive properties of collagen by the incorporation of nanoparticles with improved outcomes of CTE	Bonfrate et al. (2017)
		Magnetic Alginate (Alg.) hydrogel scaffolds	*In vitro* assessment	Cardiac tissue engineering	Fabrication of proficient pre-vascularized constructs potential for transplantation applications	Sapir et al. (2012)
		Polyethylene glycol (PEG) diacrylate magnetic nanoparticles hydrogels	*In vitro* assessment	Cardiac muscle cells engineering	Development of sophisticated platforms for drug delivery and actuation activities for CTE purposes	Vannozzi et al. (2018)
		-Cryogels based on Gelatin methacrylate (GelMA) and elastin adapted with carbon nanotubes (CNTs) and magnetic nanoparticles (MNPs)	- *In vitro* assessment	-Cardiac tissue engineering	-Enhanced engineering of the infarcted heart tissue	Pardo et al. (2021)
	Pressure-responsive hydrogels	Polymer polyaniline (PAni) hydrogel	*In vitro* (cardiomyocytes culture)	Supports cardiomyocyte organization into a spontaneously contracting system	The composites improved cardiac cell organization into a freely contracting structure with potential application in CTE	Chakraborty et al. (2018)
		-Cyclodextrin-Hyaluronic acid (CD-HA) and Adamantane -Hyaluronic (Ad-HA) hydrogels	-Ex vivo (porcine cardiac tissue)	-Cardiac tissue engineering	-Amelioretd CTE with improved restoration of the cardiac functions	Chen et al. (2017b)
	Ultrasound/acoustic-responsive hydrogels	Silk sericin (MSS)-Fe_2_O_3_nanocomposite hydrogels loaded with secretome (Sec) biomolecules (Sec@MSS)	*In vitro* (cardiomyocytes culture)	Reduction of the Doxorubicin (DOX) induced cardiotoxicity in human stem cell-derived cardiac muscle cells	Sec@MSS are promising and potent platforms for application in CTE	Zhang et al. (2021a)
		Heparin-binding based, Gd(III)-tagged PEG hydrogels	*In vivo* (mouse myocardium)	To deliver and monitor cardiac progenitor/stem cell engraftment for implantation	Heparin-binding based, Gd(III)-tagged PEG hydrogel systems presented a tailored cell delivery and potential to assess the transplanted materials for CTE	Speidel et al. (2017)
Chemical stimuli-responsive hydrogels	PH-responsive hydrogels	Poly-N-isopropyl-acrylamide- Butyl acrylate- Propyl-acrylic acid (PNIPAAm-BA-PAA) composite hydrogels	*In vivo* (rat MI model)	Improvement of the angiogenesis in infarcted myocardium	Enhanced angiogenesis and sustained topical delivery of growth factors with restored cardiac functions	Garbern et al. (2011)
		PNIPAAm with mono carbon nanotubes hydrogel entrapping stem cells	*In vivo* (rat MI model)	MI treatment	Improved cardiac tissue engineering	Peña et al. (2018)
		Hydrogen bond crosslinked ureido-pyrimidinone group to PEG	*In vivo* (pig MI model)	MI treatment	Improved delivery of growth factors and ameliorated CTE	Bastings et al. (2014)
		NIPAAm hydrogel cross linked with Di(ethylene glycol) divinyl ether (DEGDVE) [p (NIPAAm-co-DEGDVE)]	*In vitro* assessment	Cardiac Tissue Engineering	Enhanced drug release abilities of the hydrogel with potential promises in CTE	Werzer et al. (2019)
	Ionic strength-responsive hydrogels	Iron-Dopamine-gelatin (GelDA)-Dopamine-polypyrrole (DA-PPy) (Fe-GelDA and DA-PPy) composite hydrogels	*In vivo* (rat MI model)	MI treatment	Pronounced enhancement of the cardiac function restoration with improved angiogenesis	Wu et al. (2020)
		Polypyrrole-Chitosan (PPY-CH) hydrogel	*In vivo* (rat MI model)	Prevention of heart failure	-Improved cardiac functions anddeclined arrhythmia following MI	He et al. (2020)
		Self-healing ionic hydrogel (POG1) with biocompatiblepolyacrylic acid (PAA)	*In vivo* (rat MI model)	MI repair	Reduced cardiac remodeling and enhanced restoration of the heart functions after MI	Song et al. (2021b)
Biological stimuli-responsive hydrogels	Enzyme-responsive hydrogels	Matrix metalloproteinases (MMP-2) and elastase combined with Proline-Leucine-Glycine-Leucine-Alanine-Glycine (PLG|LAG) polypeptides to form biopolymer hydrogels	*In vivo* (rat MI model)	MI treatment	Ameliorated cardiac tissue engineering abilities after MI	Carlini et al. (2019)
Physical stimuli-responsive hydrogels		MMP-injectable hydrogels utilizingHyaluronic acid (HA)	*In vitro* assessment	MI treatment	Promising enzyme-responsive platforms for CTE	Li et al. (2022a)
		Recombinant protein glutathione-S-transferase (GST)-TIMP-bFGF by combining bFGF, MMP-2/9-degradable -Proline-Leucine-Glycine-Leucine-Alanine-Glycine (PLG|LAG) peptide, (TIMP), and GST entrapped in a GSH-modified collagen (Col) hydrogel (GST-TIMP-bFGF/collagen-GSH) hydrogels	*In vivo* (rat MI model)	Growth factor delivery	GST-TIMP-bFGF/collagen-GSH hydrogels could enhance the angiogenesis and reduce remodeling with improved delivery of growth factors	Fan et al. (2019a)
	Antigen/antibody-responsive hydrogels	Sulfated glycosaminoglycan-like ECM-mimetic injectable collagen (Col) hydrogel loaded with Artificial apoptotic cells (AACs) and vascular endothelial growth factor (VEGF)	*In vivo* (rat MI model)	MI treatment	Increased neovascularization at the site of MI with marked enahncement of the heart functions after MI repair	Zhang et al. (2021c)
		Magnetic basic structure nanoparticles (Fe_3_O_4_-SiO_2_) hydrogel augmented with hydrazine hydrate and aldehyde-PEG to improve antibody conjugation (Fe_3_O_4_@SiO_2_-PEG)	*In vivo* (rabbit and rat models of MI)	MI treatment	Reduced infarct size and enhanced ventricular functions with ameliorated neovascularization	Liu et al. (2020)

### 5.1 Physical stimuli-responsive hydrogels

#### 5.1.1 Temperature-responsive hydrogels

**FIGURE 4:**
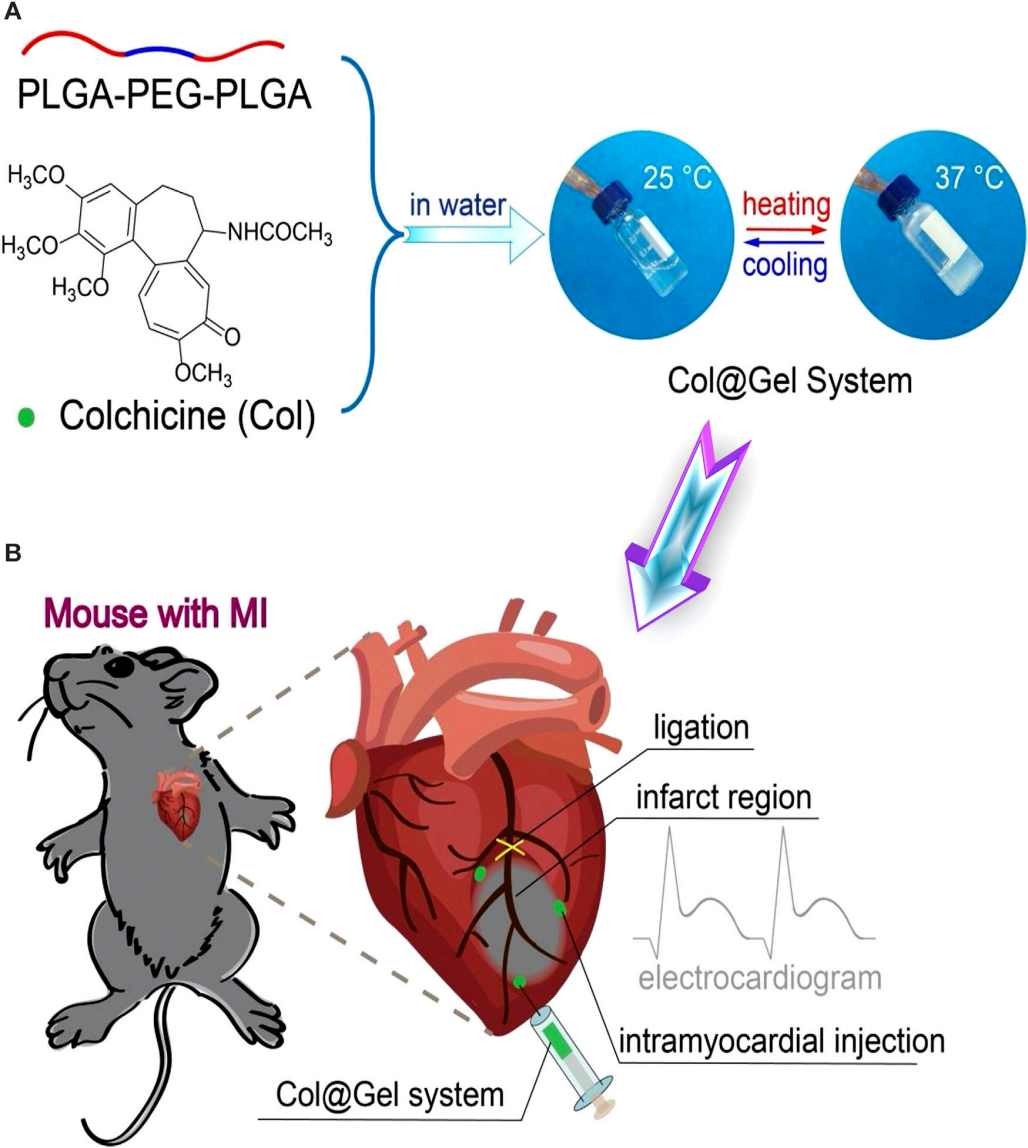
Schematic illustration of temperature-responsive (Col@Gel) hydrogels for cardiac tissue engineering. **(A)** Fabrication scheme of the hydrogel networks with reversible sol-gel phase transition responsive to the thermal stimuli. **(B)** The fabricated smart hydrogels were employed for the regeneration of the induced myocardial infarction via ligation of the left anterior descending coronary artery (LAD). Copyright © 2020, The Royal Society of Chemistry. Replicated with permission from (Chen et al., 2020b).

**FIGURE 5:**
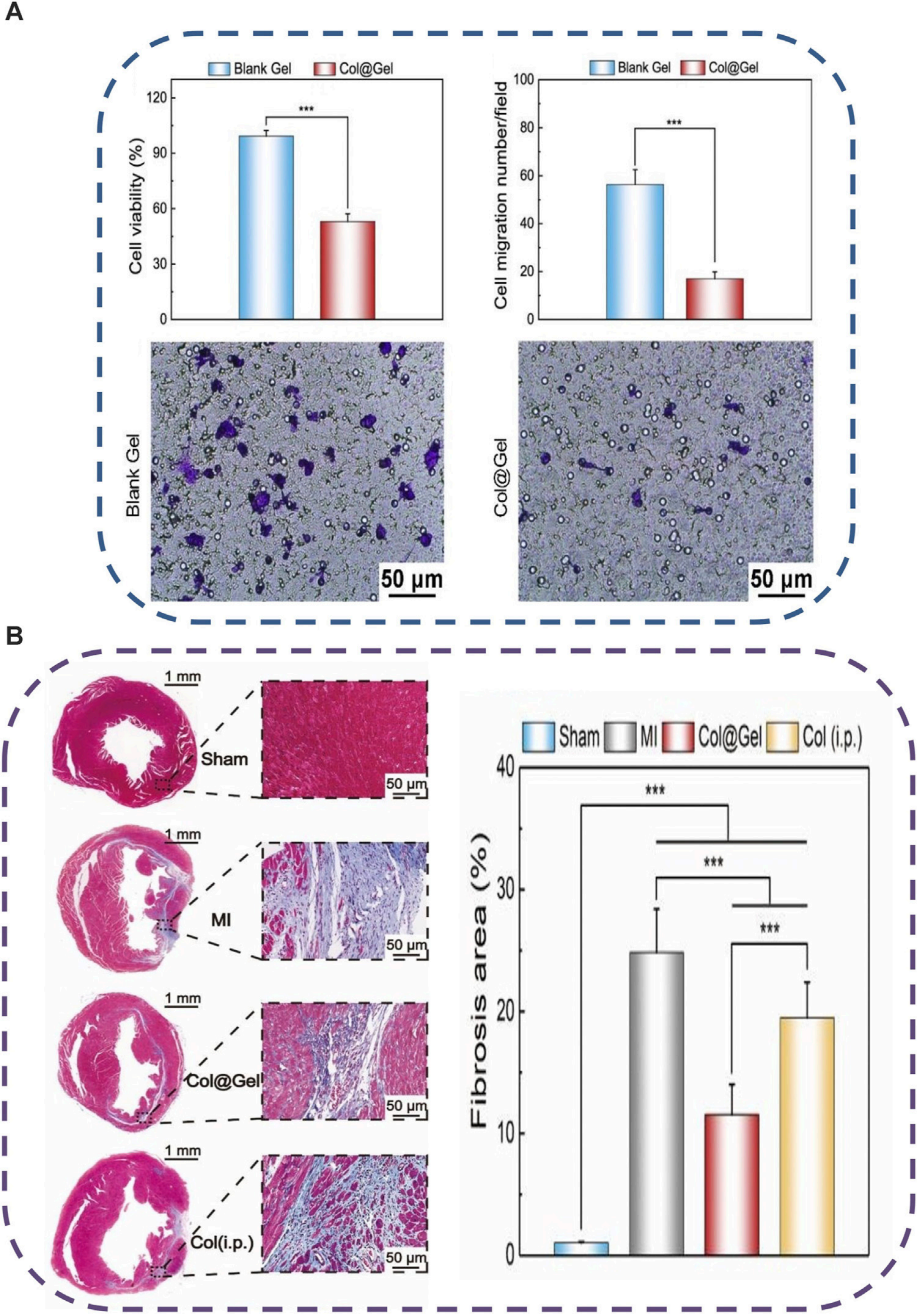
Schematic illustration of the feasibility of the fabricated thermo-sensitive (Col@Gel) hydrogels for cardiac tissue engineering. **(A)** *In vitro* inhibition of the viability and the migration of Raw264.7 macrophages. **(B)** *In vivo* injection of the Col@Gel systems ameliorated the cardiac inflammatory response and hindered myocardial fibrosis. Copyright © 2020, The Royal Society of Chemistry. Replicated with permission from (Chen et al., 2020b).

Thermo-induced hydrogel uses change in temperature as the only trigger for their gelling tendency, with no other external influences. Temperature distributions in various bodily areas vary. Particularly, it was discovered that cardiac lesions have a temperature that is distinct from the surrounding area, opening the door for targeted drug administration or controlled release of drugs utilizing temperature-sensitive hydrogels that have a phase shift (Guan et al., 2021b). These hydrogels are appealing for biomedical applications because they can change to semisolid form *in situ* under physiological circumstances and are easy to administer (Bellotti et al., 2021). Poly (N-isopropylacrylamide) (PNIPAAm) hydrogels have been widely used as delivery payloads for a variety of treatments. However, due to their poor power to promote encapsulated cell growth, they showed inferior bioactivity for encapsulated cells (Chen et al., 2013). Xia and others effectively inserted single-wall carbon nanotubes (SWCNTs) into the base PNIPAAm hydrogel, resulting in a thermo-responsive SWCNTs-modified PNIPAAm (PNIPAAm/SWCNTs) hydrogel with improved cytocompatibility (Li et al., 2014). They could test the PNIPAAm/SWCNTs hydrogel system’s bioactivity in brown adipose-derived stem cells (BASCs) and its effectiveness in delivering BASCs to the MI site. The PNIPAAm/SWCNTs hydrogels not only showed significant-high bioactivity to encapsulated BASCs *in vitro*, with enhanced cell proliferation and adhesion, but they also demonstrated a satisfactory capacity to deliver the encapsulated BASCs to the infarct myocardium *in vivo*, with enhanced engraftment of seeded cells at the MI site. In another work, an electroconductive gold nanoparticle (GNP)-loaded CH thermal-induced hydrogel for cardiac repair was explored. The electrical connection between stem cells and neighboring cardiac cells could be increased by raising the concentration of GNPs (Baei et al., 2016). A PNIPAM thermo-induced hydrogel containing PLGA-encapsulated PVP/H_2_O_2_ microspheres was used to heal cardiac cells following MI in another investigation. After 4 weeks of injection in the MI location, cardiac cells showed lower transforming growth factor expression, and cardiac fibrosis was decreased, indicating that myocardial cells were healed due to oxygen uptake (Fan et al., 2018). Another thermo-induced hydrogel made of gellan gum and rGO has been investigated. The findings of the 3-(4,5-dimethylthiazol-2-yl)-2,5-diphenyl-2H-tetrazolium bromide (MTT) experiment showed that gellan gum thermos-induced hydrogels comprising 1% and 2% rGO were not cytotoxic. Gellan gum/2 percent might be a good option for mending and recovering infarcted cardiac tissue, according to these findings (Zargar et al., 2019). Besides the numerous desirable features of chitosan polymer (Nomier et al., 2022; El-Dakroury et al., 2023), it was exploited for MI therapy, forming a chitosan (CH)/dextran (DEX)/β-glycerophosphate (β-GP) parenteral thermo-induced hydrogel occupied with umbilical cord mesenchymal stem cells (UCMSCs). UCMSCs develop towards myocardial and have significant potential for clinical applications of cardiac repair, according to the expression of cardiac markers cTnI and Cx43 and signaling pathways p-Akt and p-ERK1/2 (Ke et al., 2020). In another study different quantities of beta-glycerophosphate (-GP) and different kinds of hydrolyzed collagen HC, as well as CH, were used to synthesize thermo-induced hydrogels. The hydrogel showed cell viability of more than 75%. These properties make it appropriate for injectable and easy to perform cardiac therapeutic uses, in which cells might be trapped and directed to the wounded spot of the heart muscle via the porous structure. Based on the gelation time and biocompatibility results, it can be inferred that these hydrogels have good physical-chemical and biological properties, making them ideal prospects for TE treatment modalities against infarcted or ischemic cardiac tissue damage (Orozco-Marín et al., 2021). An injectable, thermo-induced hydrogel of poly (lactide-co-glycolide)-poly (ethylene glycol)–poly (lactide-co-glycolide) (PLGA–PEG–PLGA) was created and mixed with colchicine (Col) to form Col@Gel network (Figure 4A). The injectable solution formed *in situ* gel at 35°C and remained in a gel form at body temperature (37°C) (Figure 4B). *In vitro* evaluation of the smart hydrogel system revealed that they reduced the viability and the migration of the Raw264.7 macrophages which are the most vigorous inflammatory cells present in the MI (Figure 5A). The injection of this hydrogel ameliorated cardiac inflammatory response effectively, hindered myocardial necrosis (Figure 5B), improved cardiac performance, and enhanced mouse viability in an animal model of MI without inducing severe cytotoxic effects, as seen after administration of conventional Col solution (Chen et al., 2020b).

#### 5.1.2 Light/photo-responsive hydrogels

Light is an excellent stimulator for polymer shape alteration. Light allows for temporal and spatial modulation and distant manipulation of polymer. The quantity of energy bending deformations can be well-adapted by adjusting various illumination parameters including intensity of light, length of exposure, and wavelength. Furthermore, controlled light irradiation or changing polarization direction may be used to create well-defined and complicated 3D hydrogel structures (Jiang et al., 2020). UV-induced hydrogels and visible photo-induced hydrogels are the two types of photo-induced hydrogels. Visible light, apart from UV light, is readily available, inexpensive, safe, and clean, yet simple to use (Guan et al., 2021a). Materials that respond to light can be utilized to modify cells, particularly neuronal and cardiac cells. Inorganic nanoparticle hydrogel can stimulate a single cell by photothermal or optical stimulation employing advanced light-controlled systems (Lee and Gaharwar, 2020). A study performed by Memic et al. demonstrated that the physiological functioning of the brain and cardiac tissues was controlled using near-infrared (NIR)-sensitive polydopamine NPs. By subjecting the cells to polydopamine NPs encapsulated in collagen foam and thereafter illuminating the NIR light source on the NP-foam mixture, the electrical activity of both types of cells was regulated. Photo-induced NPs were placed in highly porous hydrogel frameworks that are physiochemically and physiologically adjustable. The highly porous design of the photo-induced hydrogel can improve cell movement (Memic et al., 2019). In another study, poly (2-alkyl-2-oxazoline) (POx) derivative was used for the preparation of photo-induced hydrogel. It was shown that the cell-degradable characteristics of the resultant POx hydrogels allow for the control of cell outgrowth in 3D matrices. In partly cell-degradable POx hydrogels, the transcription of pro-angiogenic genes was elevated. It was demonstrated that the epicardial insertion of MSC-loaded POx hydrogels improved the restoration of cardiac function in arteries and heart walls due to the outstanding tissue adhesion characteristics of thiol-ene polymerized hydrogels (You et al., 2021). Another research found that incorporating carbon nanotubes (CNTs) into a photo-cross-linkable gelatin Methacrylate (GelMA) hydrogel can result in high-performance cardiac scaffold materials. In comparison to pure GelMA, the cardiac cells in CNT-GelMA were found to be elongated, while F-actin fibers were shown to be viable and more homogenous. Synergistic beating activity can be generated by both CNT-GelMA and pure GelMA. The CNT-GelMA, on the other hand, demonstrated a beating frequency average that was both more steady and more consistent when the impulsive beating rhythms were collected over 6 days (Shin et al., 2013). Reduced graphene oxide included in GelMA photo-induced hydrogels is used to create cardiac tissue constructions. The addition of rGO to the GelMA matrix improves the material’s conductance and mechanical behavior dramatically. Furthermore, relative to GelMA hydrogels, cells cultivated on rGO-GelMA scaffolds demonstrate improved biological functions such as cell survival, proliferation, and development. During the rGO-GelMA hydrogel period, CMs exhibited improved contractility and a quicker natural beating rate (Shin et al., 2016).

#### 5.1.3 Electro-responsive hydrogels

The development of non—conducting fibrotic tissue post-CM mortality due to ischemia is a typical feature of MI. The growth of scar tissue obstructs the transmission of electric signals between CM resulting in abnormal heart rhythms and functional decompensation. Restoring electric signals within the heart allows the contraction to be resynchronized, preventing additional remodeling and ventricular dysfunction (Ghouri et al., 2018; Zhang et al., 2020a). Electro-responsive hydrogels are based on the idea that under electric stimulation, neural and cardiac tissue grows and multiplies more effectively. They must be capable of changing chemically and physically in response to electric stimulation. Electrical impulses are generated by the cardiac muscle. In the case of myocardial infarction, heart tissue gets damaged. Electro-responsive hydrogels generate essential functional properties for the development of tissue, providing an environment for cell growth. It may act as a conductive link between healthy and damaged tissues, allowing an electrical pulse to propagate throughout the infarcted area and eventually trigger synchronous muscle contraction throughout the entire heart (Carayon et al., 2020). Various hydrogels having conductive qualities, dubbed electro-induced hydrogels, have been developed to repair the heart’s electric pulses (Wang et al., 2018; Song et al., 2021a; Mousavi et al., 2021). For instance, conductive hydrogels have been made by attaching conductive polymer poly-3-amino-4-methoxy benzoic acid (PAMB) on Gt and cross-linking it using carbodiimide. The self-doped (PAMB-G) hydrogel was found to be compatible with CM *in vitro* and effective in synchronization. An *ex vivo* experiment was also conducted in the same study in which two rat hearts, one beating and the other not, were segregated by either PAMB-G hydrogel or a non-conductive hydrogel to investigate the hydrogel’s capacity to carry an electrical impulse (Gt). In this experiment, PAMB-G had a substantially greater measured improved amplitude in the non-beating cardiac muscle than the non-conductive hydrogel (Zhang et al., 2020a). PAA was mixed with oxidized alginate (OAlg.)/Gt to create a self-healing highly porous electrically conductive hydrogel. This hydrogel was shown to exhibit stable rheological characteristics, resilience, and flexibility, as well as the ability to maintain its network after or during deformation. Furthermore, CMs sown inside the hydrogel synchronized their beating and exhibited a tendency for directed development due to the hydrogel’s conductive qualities. Furthermore, the hydrogel was loaded with donor CM before being implanted rather than injected. While the hydrogel kept the cells in the heart and improved functional recovery compared to a control lacking CMs, no experiments were done to see if it might reduce arrhythmias. Song et al. proved that PAMB-G is more conductive than the non—conducting hydrogel (Song et al., 2021a). Conductive hydrogels can potentially be effective in synergistic conjunction therapy. One of the studies reported a conductive hydrogel filled with adipose-derived stem cells (ADSCs) and plasmid DNA expressing endothelial nitric oxide synthase (eNOS). The hydrogel was designed to improve electrical signal conductivity inside the infarcted heart, reduce inflammation, and stimulate nitric oxide generation and angiogenesis. To produce the conducting hydrogel, tetraaniline-polyethylene glycol diacrylate (TA-PEG) NPs were self-assembled and interacted with thiolated HA through an acrylate-thiol Michael Addition reaction (HA-SH). ADSCs elevated NOx levels in the myocardium and, as a result, stimulated angiogenesis, which improved recovery (Wang et al., 2018; El-Dakroury et al., 2022). Gold nanoparticles (GNPs) in chitosan–glycerol phosphate (CH-GP) gels have also proven to mimic the electromechanical capabilities of the heart without sacrificing thermosensitivity. Comparative to cells loaded on pure CH hydrogels, MSCs loaded on the CH–GP–GNP hydrogels demonstrated increased cardiomyogenic development (Baei et al., 2016). Poly (pyrrole) (PPy), an electro-responsive substance, was integrated into acid-modulated silk fibroin to create an electron-induced hydrogel polymer substrate. The sarcomere size and Z-bandwidth of CM cultured on this substrate increased, as well as the expression of cardiac-specific genes. Carbon nanotubes have also been studied extensively as conductive substances for cardiac engineering applications (Martinelli et al., 2018; Tsui et al., 2018; Zhao et al., 2020).

#### 5.1.4 Magnetic-responsive hydrogels

Magnetic-responsive hydrogels are constructed by incorporating magnetic nanoparticles (MNPs) in the hydrogel that are triggered by an exterior magnetic force to obtain the desired reaction in the environment (Municoy et al., 2020, Abd Elkodous et al., 2021). The number of nanoparticles incorporated in the hydrogel affects the magnetic characteristics of the hydrogel, the release behavior of the loaded medications, and the sensitivity of the cultured cells (Lin et al., 2020). MNPs can be in the form of oxides (for example, iron oxides), metallic (for example, nickel, iron, and cobalt), or plated oxides and metallic nanoparticles (Municoy et al., 2020). For cardiac engineering, scaffolds must mimic the mechanical properties of the native tissue, provide signals for cell aligning and elongation to mimic certain contractile characteristics, and permit electrical conductivity. Conductive polymers such as polypyrrole (PPy) or polyaniline, as well as noble metals for triggering by external magnetic stimuli (Pardo et al., 2021). MNPs for cell stimulation in cardiac-engineered constructions at a distance were utilized by co-precipitated magnetite MNPs combined with freeze-dried Alg. Nanoporous hydrogels. The scaffolds were inoculated with bovine endothelial cells of the aorta, and a weak AFM (1.5 mT, 40 Hz) was applied using Helmholtz coils. The stimulation of magnetic scaffolds to AFM stimulated the formation of early capillary-resemble structures in endothelial cells, replicating the biological structure of real heart tissue (Sapir et al., 2012). In a study performed by Bonfrate et al., magnetic Fe_2_O_3_ nanoparticles was produced by co-precipitation were inserted into collagen hydrogel film for cardiac TE scaffolds. It was feasible to obtain micropatterns of MNPs within the collagen matrices by using external magnetostatic fields (created by two parallel magnets or current wire configurations). This method enables the conductivity of the specified scaffolds to be increased without the use of highly conductive materials. The scientists highlighted the relevance of anisotropy in accurately mimicking the electroconductive characteristics of cardiac tissues, resulting in the development of a viable tool for triggering stem cell cardiac differentiation (Bonfrate et al., 2017). Another relevant study demonstrated PEG diacrylate hydrogels having two stacked sheets modified with widely viable MNPs as prospective cardiac and muscular TE scaffolds. The sheets were bent into 3D tubes and CMs were implanted within, resulting in high adhesion as well as survivability, and preservation of contractility over 7 days (Vannozzi et al., 2018). Another CTE, magnetically induced cryogels based on GelMA and methacrylate adapted with CNTs and MNPs were explored. Under the externally applied magnetic fields, the resulting scaffolds coupled exceptional elasticity, plasticity, and strain conductivity, making them ideal options for use as controlled conductive actuators for the heart (Pardo et al., 2021).

#### 5.1.5 Pressure/mechano-responsive hydrogels

After myocardial infarction, there are changes in the heart that led to higher filling pressures (Fan et al., 2020). Pressure-responsive hydrogels have made great advancements in the biological field (Zhang et al., 2020b). They are a special sort of hydrogel that has a high sensitivity, a lot of flexibility, and a lot of repeatability, enabling them to be employed in cycling and single-pressure measurements. Character modifications in these hydrogels are common in reaction to external stimuli. The strength of the stimulus and the impact of external stressors may be determined by examining system parameters (Pinelli et al., 2020). The capacity of these hydrogels to respond to recognized pressure stimuli is particularly noticeable in highly elastic hydrogels. Chakraborty et al. Created a conducting pressure-responsive hydrogel using a fortified dipeptide as a supramolecular gelator, owing to its inherent biocompatibility and outstanding gelation capabilities, along with the conductive polymer polyaniline (PAni), which has been polymerized in the site. The hybrid hydrogel is physically stiff, and the rigidity may be adjusted by varying the peptide content. The hydrogel has ohmic conduction, pressure responsiveness, and, perhaps most crucially, self-healing properties. The hydrogel’s innate conductivity may be restored owing to its self-healing ability after the macroscopic separation of its block. Cardiomyocytes cultured on the hybrid hydrogel have a high cell survival, indicating that it is non-cytotoxic. The hydrogel’s combination of properties allows it to be used for a dynamic range of pressure sensing as well as a conductive interface for electrogenic heart cells. Cardiomyocyte organization into a naturally contracting system is aided by the hybrid hydrogel (Chakraborty et al., 2018). Another study created a pressure-responsive hydrogel by combining HA either with -cyclodextrin (CD) or adamantane (Ad) to form CD-HA and Ad-HA, and assembling the hydrogels thru supramolecular hydrophilic groups interaction to create shear-thinning injectable hydrogels in an animal model with MI (Chen et al., 2017b).

#### 5.1.6 Ultrasound/acoustic-responsive hydrogels

**FIGURE 6:**
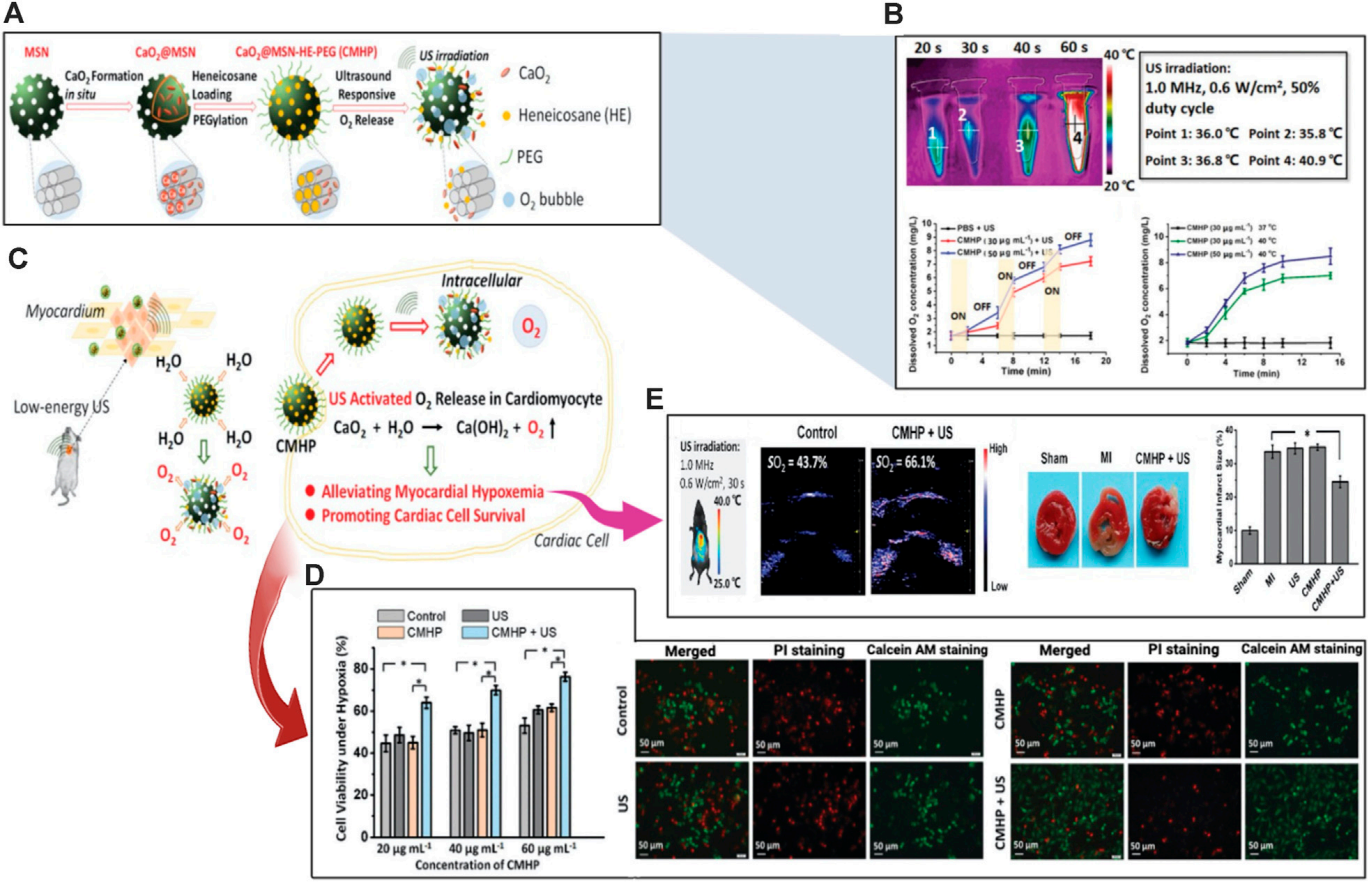
Schematic illustration of ultrasound/acoustic-responsive CaO_2_@MSN-HE-PEG (CMHP) nanosystems exploited for heart tissue engineering. **(A)** Synthesis of the US-sensitive CMHP. **(B)** Evaluation of CMHP for US-triggered hyperthermia and US-sensitive release of oxygen. **(C)** Proposed mechanism of US-responsive water diffusion and oxygen release to enhance myocardial survival. **(D)** Improved survival of the cardiac cells under hypoxic circumstances. **(E)** Enhancement of cardiac tissue engineering. Copyright © 2020, The Royal Society of Chemistry. Replicated with permission from (Fu et al., 2020).

Myocytes, which make up the majority of muscle tissue, are separated into three types: smooth, skeletal, and cardiac muscles. Together, they work to maintain the homeostasis of all tissues. Each kind is strongly related to neurological tissue. Biological cardiac engineering techniques mimic tissues using materials dispersed with isotropic tissues. The use of 3D-printed tissues is currently constrained due to resolution limitations, and these created tissues typically lack synchronization in mechanical activities and systematic consistency. But an easier and more effective technique is provided by ultrasonic hydrogel (Han et al., 2023). Attributed to its efficiency and non-invasive feature, the US has widely employed medicine to image inner tissues, allowing for illness diagnosis and treatment. US waves, like other external stimuli, can be used to remotely initiate on-demand medication administration and enable the development of novel therapeutic platforms with deep tissue penetration (Lavrador et al., 2021). For example, US-responsive systems have the potential to deliver bioactive therapeutics in a temporally controlled way in a variety of ways, such as pulsatile or uni-directional burst release, and over extended periods following *in vivo* application (Manouras and Vamvakaki, 2017). Ultrasound-induced acoustic energy could upset the self-assembly equilibrium of nanocarriers such as lipid nanoparticles, polymeric micelles, and nanocapsules, causing them to disassemble. Silica-based nanoparticles have been widely investigated in US-based applications, benefiting from improved acoustic cavitation and permitting the loading of US disturbed mesopores. Hydrogel networks formed with non-covalent connections can also be spontaneously fragmented with the application of ultrasonic waves, in addition to US-responsive nanomaterials (Zhou et al., 2017b). Another US disrupted injectable hydrogel architecture mixed with nanoparticles and secretome molecules was synthesized to reduce doxorubicin (DOX)-induced apoptosis CMs. A biocompatible silk sericin (MSS) matrix form including Fe_2_O_3_ nanoparticles was synthesized and employed as an injectable vehicle for secretome for cardiomyocyte metabolism *in vivo*. The secretome-encapsulated Fe_2_O_3_ -Silk sericin (Sec@MSS) hydrogel was recommended as a therapy for heart problems (Zhang et al., 2021a). Another multifunctional US-guided hydrogel consists of a PEG network linked with Gd III) peptide allowing MRI to monitor the hydrogel’s localization and retention *in vivo*. Heparin-binding peptide (HBP) sequencing in the cross-linker design to customize the polymer for cardiac purposes, the formed gel has mechanical characteristics similar to those of heart tissue. The metabolic activity of luciferase-expressing cardiac stem cells (CSC-Luc2) contained inside these gels was sustained lasting for 14 days *in vitro*. CSC-Luc2 persistence in the mouse heart and hind limbs was enhanced by 6.5 and 12 times, respectively, after encapsulation in HBP hydrogels (Speidel et al., 2017). In a recent elegant study, Fu H. et al., (Fu et al., 2020) designed ultrasound-sensitive nanomaterials to generate oxygen at the infarct site (Figures 6A, C). Moreover, they reduce the hypoxemic myocardial milieu to save the cardiac cells following AMI. The construction of the thermosensitive substance heneicosane and polyethyleneglycol was done after the synthesis of calcium peroxide in the mesopores of biocompatible mesoporous silica nanoplatforms. It was possible to achieve US-responsive diffusion of water and release of oxygen thanks to the phase change of heneicosane that was caused by the slight hyperthermia brought on by US irradiation (Figure 6B). The cardiac cell survival under hypoxic circumstances was markedly enhanced (Figure 6D). After an AMI, the US-activated oxygen release greatly reduced hypoxia and aided in the reduction of oxidative stress. As a result, the damage to the infarcted myocardial tissue was reduced (Figure 6E). This nanosystem for US-activated oxygen production might offer an effective AMI therapy option and keeps the do open for future integration of this strategy to produce more sophisticated US-sensitive nanocomposite hydrogels for CTE applications.

### 5.2 Chemical stimuli-responsive hydrogels

#### 5.2.1 pH-responsive hydrogels

pH is a commonly changed biological trait in pathologic conditions including inflammatory disease and tumors as well as anatomical regions including different parts of gastrointestinal tract. PH-responsive hydrogels can readily vary their properties to promote site-specific drug delivery. The prevalent methods for creating pH-responsive hydrogels are the inclusion of a covalent bond that is susceptible to breaking down by changing pH or the employment of polyelectrolytes, having several ionic terminal groups that absorb or give protons in reaction to pH variations in the environment (Kim et al., 2014). Significant pH variations may be seen in various body regions both during healthy function and when a disease is present as myocardial ischemia and myocardial infarction (Jiang et al., 2022). Changes in pH can be exploited as triggers for medication delivery to areas of local acidosis, such as those observed in myocardial ischemia (pH 6,7) (Saludas et al., 2017). Several attempts have been done to create pH-responsive hydrogels utilizing different copolymers that respond to acidic pH, including carboxylic acid-derivative as propyl acrylic acid, and ethyl acrylic acid (Yin et al., 2006), methacrylic acid (Lackey et al., 1999), or acrylic acid (Chen and Hoffman, 1995). A study performed by Yin, et al., demonstrated the development of an injectable pH-responsive hydrogel comprising poly-N-isopropyl-acrylamide (PNIPAAm), butyl acrylate (BA), and propyl-acrylic acid (PAA). At pH 7.4, this polymer is a liquid, but at 37°C and pH 6.8, it transforms into a gel. It was assumed that this polymer’s tendency to develop a reversible gel in medium acidic environments permits it to behave as a depot structure for the discharge of angiogenic growth factors to the ischemic myocardial tissue, and then to promote polymer disintegration and removal when the tissue recovered to physiological pH (Yin et al., 2006). In an attempt to improve the previous hydrogel, reversible fragmentation addition chain polymerization was used for hydrogel synthesis. This technique improved the physical strength of the gel and increased the retention time of the growth factor into infarcted rat myocardium by 10-fold promoting 30e40% raised capillary and arteriolar densities (Garbern et al., 2011). PNIPAAm hydrogel has also been used to permit the injection of mono CNTs to contain entrapped stem cells derived from adipose cells into the heart of a rat MI model using. It was discovered that the hydrogel improved cell incorporation and provided a therapeutic effect (Peña et al., 2018). In another study, the catheter-injectable hydrogel was created by linking the hydrogen bonding ureido-pyrimidinone group to PEG through alkyl-urea spacers (Bastings et al., 2014). In a recent study, multiple drugs including phenytoin, indomethacin, and clotrimazole were slipped on glass using polymerization techniques to construct pH-induced hydrogel, and then *in vitro* release study was conducted, where the blockage of release was measured *versus* different pH (Werzer et al., 2019).

#### 5.2.2 Ionic strength-responsive hydrogels

Ionic strength is a frequent and readily regulated stimulus, hence ionic strength-induced hydrogels might be useful in a variety of situations. Ionic-strength-induced hydrogels are frequently made from ionizable polymers that swell differently in water and an electrolytic solution. The degree of ionization and density of charge of the hydrogels have a significant impact on the surrounding solution’s expansion and shrinkage behaviors (Xiang et al., 2018). The Purkinje fibrous materials in the myocardium, that conduct electricity, make up the heart and are in charge of transmitting electrical impulses. One of the leading causes of mortality globally is myocardial infarction, which irreversibly destroys heart tissue. Ionic-responsive hydrogels offer a 3D environment for cell development, and the addition of metal ions produces crucial functional characteristics for tissue formation. The use of ionic-responsive hydrogel is a particularly promising technique because it may serve as a conductive bridge between healthy and damaged tissues, causing an electrical pulse to spread throughout the infarcted area and eventually inducing the synchronous muscle contraction in the entire heart (Janarthanan et al., 2021). Using Fe^3+^-induced cationic coordination, a uniform hydrogel matrix was formed combining dopamine-gelatin (GelDA) and dopamine-polypyrrole (DA-PPy). As cardiac patching, this conductive hydrogel increases electrical signal conductivity in infarcted myocardium (Xiang et al., 2018). In another study, PPy-chitosan hydrogel (PPy-CH) hydrogel was employed to investigate the role of ionic strength-induced hydrogels in avoiding heart failure. The findings showed that ionic strength-induced hydrogels might improve electrical conductivity to synchronize cardiac contractions by lowering electrical resistance in the infarcted region, laying the groundwork for ionic strength-induced hydrogels to be used in myocardium infraction MI therapy (He et al., 2020). A PPY-CHI hydrogel was also created and employed for MI treatment. The findings showed that, as compared to conventional hydrogels, the ionic strength-induced hydrogels efficiently increased Ca^2+^ conduction in rat CMs *in vitro*, depressed the QRS interval, and improved electrical pulsed signal transmission and heart function upon MI (Mihic et al., 2015). For the management of MI, ionic strength-induced hydrogels have been progressively utilized. Ionic strength-induced hydrogel choline-bio ionic liquid (Bio-IL) embedded in a GelMA hydrogel, increases primary CMs adhesion, proliferation, and electro-modification *in vitro* (Noshadi et al., 2017). PAA was also incorporated into oxidized alginate (OAlg.)/Gt combination to create a new hydrogel (POG) with outstanding conductance and self-healing capabilities due to its side-chain carboxyl groups deprotonating into COO^−^ in the medium. The POG hydrogel also enhances CM resynchronization by participating in an efficient interaction with CMs via electrical communication. POG hydrogel has been shown *in vivo* to drastically minimize LV remodeling and recover myocardial performance (Song et al., 2021a). Another ionic strength-induced hydrogel was synthesized by dissolving gold nanorods (GNRs) in GelMA solution. When cultivated GelMA-GNR hydrogels, the ionic conductivity of the hybrid hydrogel was modified to facilitate rhythmic contraction of the CMs (Navaei et al., 2016). The ionic strength-induced hydrogel was also prepared by ultrasonically distributing CNTs into a hydrogel precursor solution. The electrical connection between CMs was greatly enhanced via this hydrogel (Pok et al., 2014).

#### 5.2.3 Redox-responsive hydrogels

**FIGURE 7:**
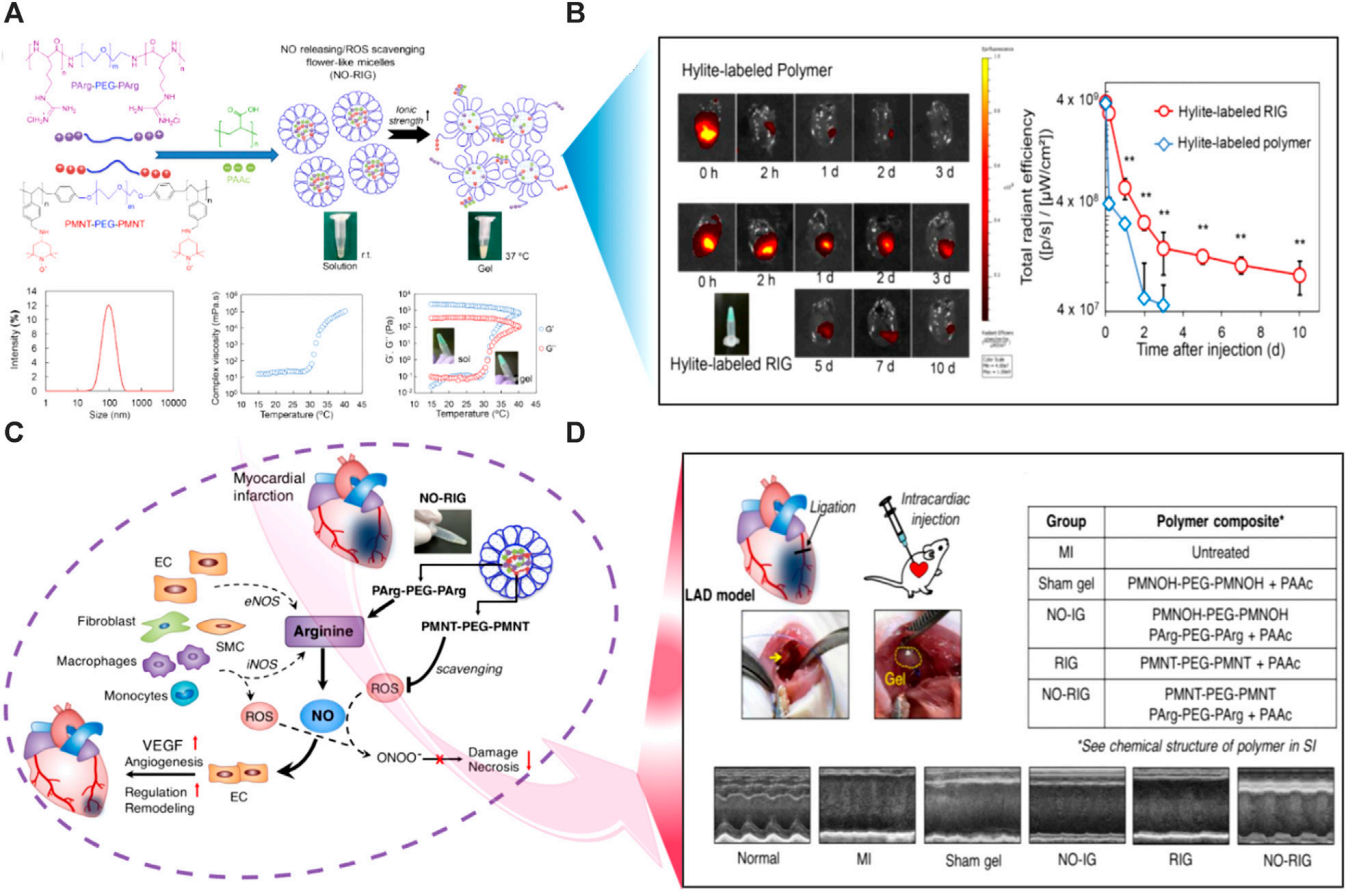
Schematic illustration of redox-responsive poly (arginine)-based nanoparticles injectable hydrogels (NO-RIG) to sustain nitric oxide (NO) release in the MI tissues. **(A)** The synthesis scheme of the NO-RIG smart hydrogels. **(B)** Assessment of the retention of NO-RIG after intramyocardial injection using a Hylite-labeled polymer. **(C)** The proposed mechanism of redox-responsiveness of the NO-RIG hydrogels with the release of NO in the damaged heart tissue to improve cardiac regeneration. **(D)** Restoration of the infarcted heart structure and consequently function was confirmed using echocardiography. Copyright © 2018, ELSEVIER Publishing Group. Replicated with permission from (Vong et al., 2018).

**FIGURE 8:**
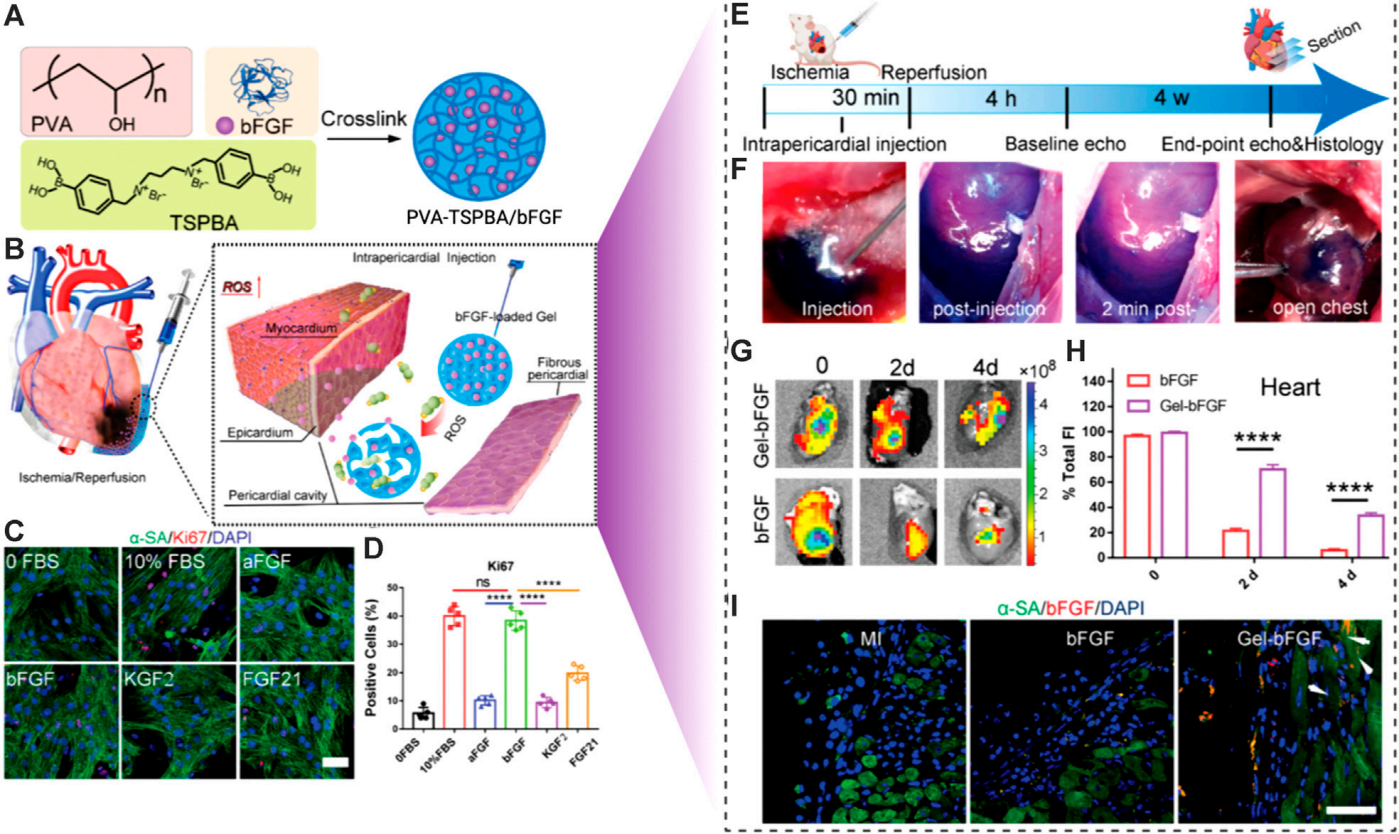
Schematic illustration of ROS-responsive smart hydrogel for the engineering of the injured cardiac tissues. **(A)** The synthesis scheme of poly (vinyl alcohol) (PVA) based PVA/bFGF hydrogel. **(B)** Mechanism of action of the ROS-responsive hydrogel following intra-pericardial injection. **(C)** Fluorescent imaging and **(D)** calculation of Ki67 positive cells indicated that the bFGF enhanced the growth and proliferation of the NRCMs *in vitro*. **(E)** Timeline of the *in vivo* study. **(F)** Intra-pericardial injection of the designed smart hydrogels. **(G)** *Ex vivo* imaging of the hearts following injection. **(H)** The fluorescence intensities were evaluated. **(I)** Fluorescent images revealed the spread of growth factors into the cardiac muscles. Copyright © 2021, Wiley-VCH GmbH. Replicated with permission from (Li et al., 2021).

A redox state is critical for a variety of everyday biological processes in humans, including cell cycle progression (cell growth, maturation, and mortality), gene expression, Calcium ions modulation, energy metabolism, immunological response, and neurogenic activity. People with cancer, cardiovascular disease, and diabetic patients all have uncontrolled redox metabolism. In a normal person, a redox reaction, particularly glutathione/glutathione disulfide (GSH/GSSG), the archetypal cellular redox couple, works continually to maintain the redox state controlled by buffering the quantity of oxygen species reaction. Reduction is a further attainable feature of the CVD environment. Disulfide and diselenide bonds are extensively investigated reduction-responsive connections that are easily dissolved by glutathione (GSH), leading to the breakdown of carriers. However, only a small number of reduction-responsive hydrogels have been assessed for the treatment of CVDs; instead, most of their research has focused on tumor imaging and therapy. In certain subcellular regions and at sick regions of CVDs, both the level of GSH and ROS are comparatively high, which are characteristic elements to produce redox states. Some redox-responsive hydrogels have been created for focused cargo delivery and/or improved CVD diagnostics in order to take advantage of this property (Li et al., 2022b). Redox-induced hydrogels can be used in a wide range of biomedical applications. Redox-induced hydrogels can be used as biodegradable frameworks for organ and tissue healing in TE. Active species including growth factors may be placed into these hydrogels and their release is activated by redox stimulation to enhance cell penetration and proliferation. Redox-responsive hydrogels, particularly those formed by *in situ* gelling methods, can be exceedingly effective for topical medication delivery (Cheng and Liu, 2017). The use of suitable polymers in the creation of redox-induced hydrogels is critical for achieving biocompatibility and desirable performance. To identify reactive oxygen species (ROS) and prevent cells from oxidizing as a result, redox-responsive hydrogels were created. In the human body, ROS, which are highly reactive substances generated during cell metabolism, are prevalent. Furthermore, despite their powerful oxidation-induced damaging action on cellular fluids, nucleic acids, and proteins (Loh et al., 2012), they make a significant contribution to cell signaling. Hence, increased generation of ROS is frequently linked to various systemic diseases and disturbances in a number of body activities (Liu et al., 2017b). ROS-responsive drug delivery devices have received much research in the treatment of cancer, immunotherapy, and gastrointestinal (GI) illnesses (Wilson et al., 2010; Lee et al., 2013; Dai et al., 2016; Chen et al., 2019). However, the low ROS concentrations in the aforementioned microenvironments prevent additional ROS-triggered drug release applications (Kim et al., 2016). In contrast, Ischemia/reperfusion (I/R) heart damage causes a quick buildup and continual creation of ROS, which may be exploited as a trigger for medication release. Vong, L. B. et al., (Vong et al., 2018) have developed poly (arginine)-based nanoparticles injectable hydrogel (NO-RIG) to sustain nitric oxide (NO) release in myocardial tissue (Figure 7A). NO-RIG hydrogels could scavenge ROS and so boost NO bioavailability. Following injection, using a Hylite-labeled polymer, the retention of NO-RIG was evaluated (Figure 7B). NO-RIG is made up of two biofunctional polymers: PArg-PEG-PArg, which produces NO by activating macrophages in inflamed tissues, and PMNT-PEG-PMNT, which scavenges ROS to boost the activity of NO. The NO delivery and redox equilibrium balance in the infarcted tissues can be stably controlled by NO-RIG (Figure 7C). The NO-RIG therapy considerably slows the course of MI and enhances cardiac functions, according to the findings. NO-RIG-treated animals showed a remarkable increase in angiogenesis. Researchers discovered that only the animals treated with NO-RIG demonstrated significant restoration of cardiac performances in both major and mild MI models (Vong and Nagasaki, 2019) (Figure 7D). Zhenhua Li and co-workers (Li et al., 2021) synthesized a basic fibroblast growth factor (bFGF)-loaded and ROS-responsive hydrogel (Gel-bFGF) and immediately injected it into the pericardial cavity as a method for heart repair as presented in Figure 8A. The reasoning for this is that bFGF will be carried by ROS-sensitive crosslinked poly (vinyl alcohol) (PVA) hydrogel, which will dissolve under ROS to release bFGF into the heart “on-demand” (Figure 8B). The influence of these growth factors was assessed *in vitro* using rat cardiomyocytes (NRCMs). The findings showed that bFGF had the maximum effect to support cell growth and proliferation indicated by the elevation of Ki67 positive cells (Figures 8C, D). Moreover, they injected the gel into the pericardium and monitored the animals over 4 weeks (Figures 8E, F). After being delivered, the hydrogel spread across the heart’s surface and instantly formed an epicardiac patch. The myocardium might be penetrated by bFGF released from the gel (Figures 8G, H, I). With improved angiomyogenesis, such intervention preserves cardiac function and lessens fibrosis in the post-I/R heart. Additionally, both in pigs and human patients, it was shown that minimally invasive injection and access into the pericardial cavity were safe and feasible.

### 5.3 Biological-responsive hydrogels

#### 5.3.1 Enzyme-responsive hydrogels

**FIGURE 9:**
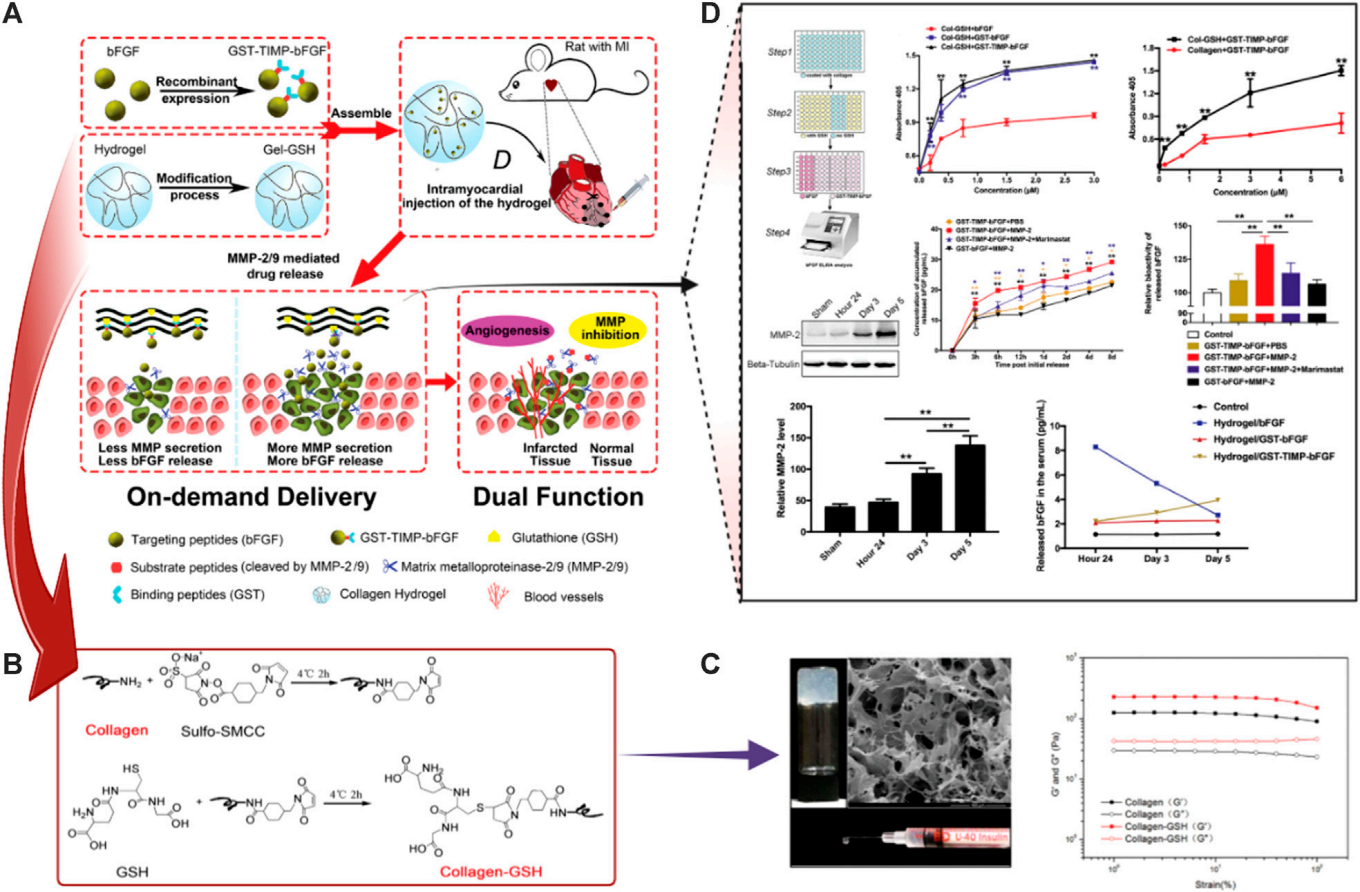
Schematic illustration of a dual function enzyme-responsive hydrogel for cardiac tissue engineering. **(A)** Fabrication scheme of GST-TIMP-bFGF/collagen-GSH MMP enzyme-sensitive hydrogels. **(B)** Conjugation of GSH on collagen using Sulfo-SMCC. **(C)** Scanning electron microscopy and mechanical characterization of the produced hydrogels. **(D)** Assessment of the binding capacity of the bFGF to the GST-TIMP-bFGF/collagen-GSH hydrogels and the MMPs enzyme-induced release profiles of these growth factors from the hydrogel. Copyright © 2019, Wiley-VCH GmbH. Replicated with permission from (Fan et al., 2019a).

Enzymes play a critical role in metabolic as well as biological functions. In cardiovascular diseases like atherosclerosis, MI, and thrombus, upregulated levels of certain enzymes were reported, which can be used as triggers for site-specific delivery and prompt release of contents at sites of action. The majority of the enzymes utilized in enzyme-responsive hydrogels include amino acid enzymes, as well as peroxidase, endonuclease, and others. Self-assembled peptide (SAP) is a versatile substance that has been successfully evaluated utilizing surgical epicardial insertion in a number of experimental MI models (Li et al., 2017). SAP spontaneously assembles into organized nanostructures via electrostatic and amphiphilic associations. Various enzyme-responsive nanocomposite hydrogels have been developed in this regard, which may be destroyed by certain enzymes to trigger or reveal bioactive components for later diagnosis or therapy. Proteases and esterases degrade short peptide fragments or esters, which are the major processes. The biological exclusivity and unique sensitivity of enzymes towards their substrates are key advantages of using enzymes to drive nanocarrier disintegration, allowing for targeted and biomaterials payload release (Li et al., 2022a). A range of enzyme-sensitive hydrogels was used to treat MI, taking into account overproduced enzymes beyond the first stage of MI. Carlini et al. created an enzyme-responsive hydrogel that flowed easily during injection and generated hydrogels *in situ* of MI in rats overexpressing matrix metalloproteinases (MMP-2) and elastase. In this scenario, cyclic self-assembling peptides were combined with a Proline-Leucine-Glycine-Leucine-Alanine-Glycine (PLG|LAG) polypeptide that may be broken by MI-related proteolytic enzymes. Due to the self-assembly of peptide sheets forming fibrils, the non-Newtonian solution of peptide solidified into viscous hydrogel when triggered by proteases in the infarcted cardiac disease (Carlini et al., 2019). Purcell et al. demonstrated MMP-injectable hydrogels utilizing HA that may be infused into the MI region and liberate rTIMP-3 (a recombinant tissue inhibitor of MMPs) since the utilized MMP-cleavable peptide (GGRMSMPV) cross-linker can be decomposed by active MMPs. When aldehyde and hydrazide functionalized HA were combined instantaneously through a double-barrel syringe, MRI confirmed hydrogel formation. In a pig MI model, intramyocardial injection of these hydrogels significantly reduced MMP expression in the damaged region, although MMP activity remained unaffected (Li et al., 2022a). Further research revealed that the localized distribution of an MMP-responsive hydrogel discharging a recombinant TIMP is a viable method for preventing negative post-MI remodeling (Purcell et al., 2018). In another research enzyme-responsive micelles hydrogel was created out of peptide-polymer amphiphiles which can be identified and destroyed by MMP-2, allowing for targeted deposition and long-term retention in MI cardiac muscle. In response to upregulated MMPs in MI cardiomyocytes, the produced nanoparticles experienced a morphological transformation from spherical substances to a network-like structure hydrogel after IV injection. The sick location can be covered with the *in situ* created scaffold for up to 28 days. Despite this, the therapeutic benefits of MMP-responsive and adaptable nanoparticles have yet to be proven (Nguyen et al., 2015). Recently, Fan, Caixia, et al. (Fan et al., 2019a) created a recombinant protein glutathione-S-transferase (GST)-TIMP-bFGF by combining bFGF, an MMP-2/9-degradable peptide PLGLAG (TIMP), and GST, which was then entrapped in a GSH-modified collagen hydrogel to create MMP-sensitive hydrogels (GST-TIMP-bFGF/collagen-GSH) for discharging of growth factors in the MI region (Figures 9A–C). The quantity of GSTTIMP-bFGF loaded in the collagen-GSH hydrogel is greatly increased by the specific binding between GST and GSH. Moreover, they showed a substantial release of these growth factors from the hydrogel platforms responsive to the MMPs enzymes (Figure 9D). During MI, the TIMP peptide that is sandwiched between GST and bFGF reacts to MMPs for on-demand release. Additionally, after a MI, the excessive breakdown of the myocardial matrix by MMPs is inhibited by the TIMP peptide, a competitive substrate of MMPs. After local injection of the hydrogel to the damaged location of MI rats, MMP-2/9 destroyed the precursor peptide TIMP, accompanied by bFGF emission, decreasing MMP activity, boosting angiogenesis, and encouraging MI healing by enhancing vascularization and relieving myocardial remodeling. According to the findings, simultaneously regulating binding and responsive release to encourage angiogenesis and lessen cardiac remodeling may be a potential strategy for treating ischemic heart disease (Fan et al., 2019a).

#### 5.3.2 Antigen/antibody-responsive hydrogels

**FIGURE 10:**
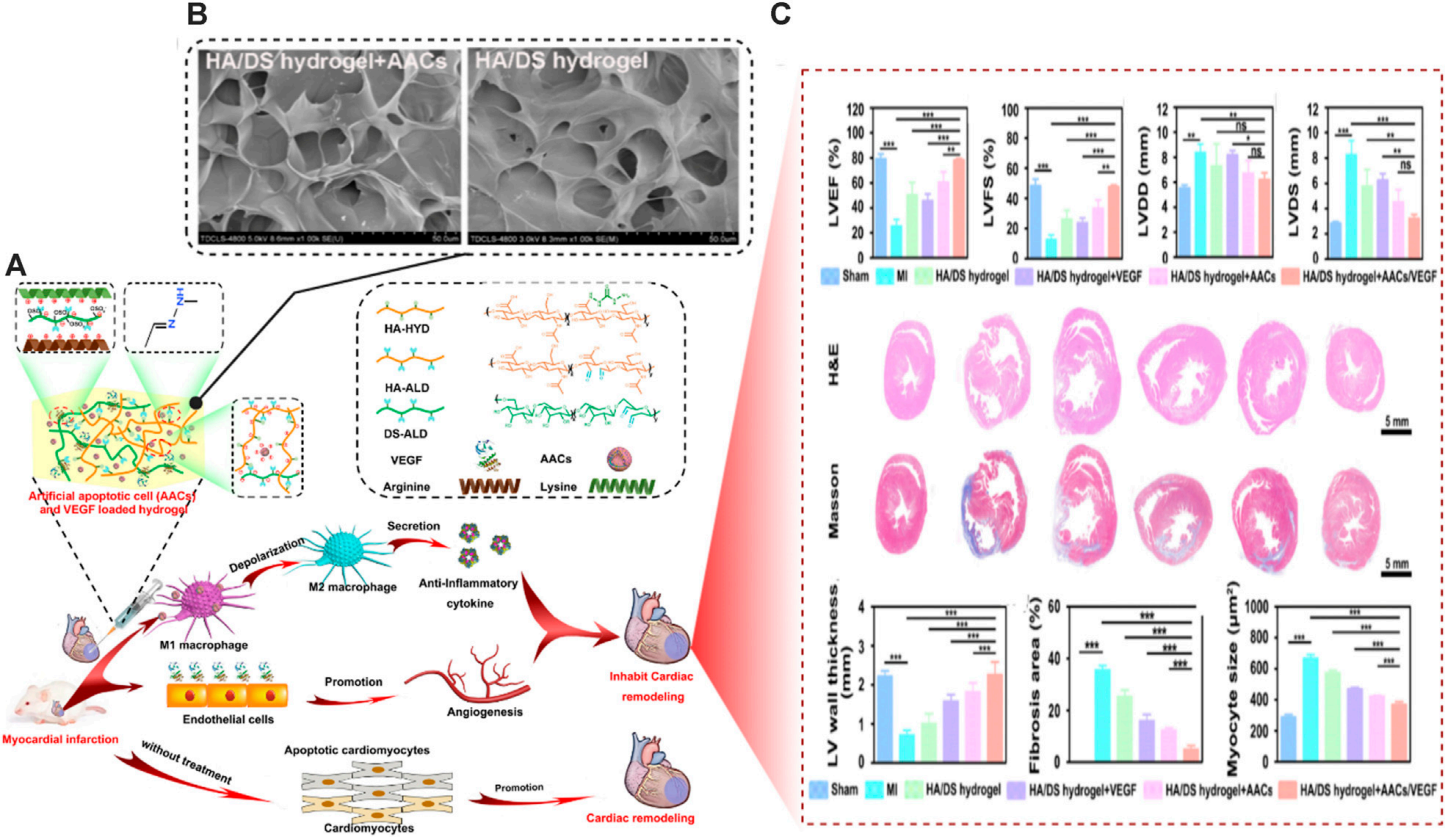
Schematic illustration of the role of smart biological-responsive hydrogels for cardiac tissue bioengineering. **(A)** Designing and responsive release of growth factors from the artificial apoptotic cells (AACs)/VEGF-loaded ECM-mimicking hydrogels for the revitalization of MI. **(B)** Scanning electron microscopy of the fabricated hydrogel platforms with and without AACs. **(C)** *In vivo* enhancement of the cardiac functions and structure assessed by echocardiography, histopathology, and gross pathological evaluations. Copyright © 2021, ELSEVIER Publishing Group. Replicated with permission from (Zhang et al., 2021c).

Materials that react to a particular biomacromolecule (such as proteins or polysaccharides) would be intriguing since they may imitate a natural response process. Materials that may expand and contract in response to a particular antigen were created specifically for this use. The antigens or antibodies can be retained inside the hydrogel through chemical coupling or polymerization or they can be added to macromers to provide reversible cross-linking within the hydrogel matrix (Acciaretti et al., 2022). Hydrogels were commonly employed to contain these chemicals because of their exceptional characteristics, such as microporous, higher binding affinity, and significant stability (Scarano et al., 2010). Artificial apoptotic cells (AACs) were created by modifying liposomes with phosphatidylserine (PS) exposed at the out layer, to replicate the activity of apoptotic cells and stimulate macrophage M2 polarization (Zhang et al., 2021c). The AACs and VEGF were enclosed in a sulfated glycosaminoglycan-like ECM-mimetic injectable hydrogel. The loaded AACs and VEGF could achieve faster and slower sustained release, which is controlled by different electrostatic interactions (Figures 10A, B). On-demand management of inflammatory immune response and increased angiogenesis was achieved by the regulated spatiotemporal administration of AACs and VEGF. The local precise release of AACs decreased the inflammatory response and lowered cardiomyocyte death in a rat model of MI with the intramyocardial treatment of an injectable hydrogel carrying AACs and VEGF (Zhang et al., 2021c) (Figure 10C). As the VEGF signaling cascade was activated by the sustained release of VEGF, revascularization was made possible in the targeted infarcted area, ultimately leading to a considerable improvement in cardiac function and reduced pathological remodeling of the left ventricle (Figures 10A, C). In a different study, magnetic basic structure nanoparticles (Fe_3_O_4_-SiO_2_) hydrogels were produced and augmented with hydrazine hydrate and aldehyde-PEG to improve antibody conjugation (Fe_3_O_4_-SiO_2_-PEG-CHO; GMNP). Exosomes were captured and damaged CMs were targeted using the nanoparticles that had been coupled with anti-CD63. Endogenous CD63-expressing exosomes were collected by anti-CD63 after IV injection, and the nanoparticles were accumulated near the region of heart damage using an exogenous magnetic field. Exosome bioavailability was increased, heart function was improved, and MI was decreased in animal models *in vivo* investigations (Liu et al., 2020). These strategies for fabricating an injectable smart hydrogel with a precisely tailored discharge of diverse growth factors to the targeted area of MI will provide outstanding platforms with great promises for the clinical treatment of ischemic heart problems.

## 6 The role of stimuli-responsive hydrogels in 4D printing

Hydrogels have grown in significance among the various materials that can be processed to exhibit a shape-morphing effect (four-dimensional (4D) printing) because of their capacity to change their shape in a reversible manner and because it is feasible to foresee and control this behavior (Piedade and Pinho, 2022). Moreover, in a process known as bioprinting, this kind of abiotic material can be printed alongside biotic materials like cells. Many trigger stimuli, including temperature, humidity, pH, concentration, ionic strength, light, and electric field, can be used to produce these changes to cause the shape-morphing property connected with the 4D effect (Lui et al., 2019). By causing varying degrees of swelling and crosslinking in various regions of the printed hydrogel, the form change may be tailored. As a result, it is feasible to model the finished part or component to specify where, how, and in which direction the shape change will take place. Hydrogels for 4D printing can be created using many materials or only one precursor hydrogel formulation. The processing methods most frequently used to create these constructions are extrusion- and light-based (Piedade and Pinho, 2022). The research of stimuli-responsive materials in 4D printing has been sparked by the maturing of additive manufacturing (AM) technology, advancements in material sciences, and improved knowledge of biological systems during the past few decades. In particular, 4D printing has the potential to be useful for a wide range of biomedical applications, including tissue regeneration, the construction of medical devices, drug delivery, and medical diagnosis (Gao et al., 2016). The ultimate goal of tissue regeneration is to design functional scaffolds to replace damaged or diseased tissues. The ideal bio-mimicking features and bio-responsiveness of synthetic materials would aid in tissue remodeling. Scaffolds with complex designs and controlled changes in their qualities over time can be successfully fabricated using 4D printing technology. Medical devices made with 4D printing can be made small and compact before implantation to lessen the size of the surgical site, and then they can expand to their final, ideal size and shape inside the body (Zarek et al., 2017). The notion of using 4D printing in CTE is still at the beginning. The rate of development in this area has been constrained by difficulties in creating printable, biocompatible, and functioning smart hydrogels. Recent advances in the development of a cardiac tissue microsystem have been made possible by the use of a range of functional inks that direct the assembly of cardiac cells and integrated sensors that provide quantitative electrical readouts of cardiac cell contractions (Lind et al., 2017). As proof of concept that such a platform might be utilized for high-throughput drug screening, the authors also carried out a number of drug experiments. These early advances in 4D cardiac printing have been more concerned with generating platforms to research heart tissue for drug screening and disease modeling rather than clinically relevant constructions. It is interesting to note that the creation of a soft-tissue robot using genetically altered cardiomyocytes has been recently investigated. In this work, researchers developed a sting-ray-shaped gadget that can move forward in water in response to optogenetic stimulation by using optogenetics technology in modified cardiomyocytes. Each layer of cardiomyocytes that made up the robot itself contributed to the sting-overall ray’s ability to move ahead (Park et al., 2016). To develop heart tissue that is clinically useful, this amount of complexity is required.

## 7 Conclusion and future prospects

To summarize, we have discussed the available options for CTE in the current review, which include the use of patches and cells, CTE approaches, scaffold-less procedures, cell assembly, ECM decellularization, and neovascularization. The employment of conventional hydrogels in CTE with the existing limitations of their use was highlighted. Then, we went over the various kind of SRHs that were exploited for CTE in great detail. A great deal of effort has gone into developing more accurate hydrogel-dependent scaffoldings for CTE applications during the last few decades. As a result, the planning, manufacturing methods, characteristics, and utility of SRHs have undergone a paradigm change. By changing the structure of hydrogels, researchers hope to increase their biological, chemical, and mechanical properties. As a result, they are among the most commonly utilized matrices in healthcare. Several attempts have been made to create SRHs for specific purposes by carefully assessing their surrounding microenvironments and leveraging these efficient improvements for future usage. The development of gels that respond to numerous stimuli and mimic the natural 3D framework is a topic that can be researched further in the future. The use of diverse polymers in the building of various hydrogels, as well as the design of new hydrogels sensitive to extrinsic stimuli, are examples of these scientific breakthroughs. Furthermore, the development of SRHs with crucial physicochemical properties that are both robust and efficient is still under investigation. Future research will focus on creating unique intelligent hydrogels with pre-programmed, even complex self-bending, twisting, and folding behaviors. Despite significant advances in hydrogel synthesis methods, biodegradation rate, microstructures, external hybridization, and immunological responses remain potential manufacturing roadblocks. As a result, more attention should be placed on designing gels that can control and decrease immune responses. The use of several solvents during the manufacture of SRHs raises the risk of toxicity. As a result, spontaneous polymer amalgamation during crosslinking is a crucial area for further research. Future study into the biodegradation of hydrogels, as well as the management of their levels of cell growth and adhesion, is necessary. Future research should concentrate on shrinking the gel matrix down to single cells, enabling cell-by-cell customization of sequencing data. This is required in order to have a better understanding of the biological subgroups in the sample. Furthermore, for smartly regulated drug delivery, changing the frequency and speed at which drugs and biomolecules are released from smart hydrogels is critical. The creation of enhanced electro-sensitive scaffolds with quicker reaction times should be the focus of future research. Platforms that respond to antigens or antibodies are great smart materials for biomedical diagnostics and CTE. Swelling-deswelling transitions are caused by either antibody or antigen. The way to fabricate smart hydrogels that react to antigen-antibody interactions has not yet been documented. As a result, further study on this subject appears to be intriguing. Furthermore, the utility of antibody-conjugated hydrogels has been demonstrated in several research. The production of SRHs with antibodies incorporated in their composition is yet a future research concern. As the design and function of SRHs improve, the cell-scaffold interactions will become more obvious. This will pave the way for new CTE goals to be fulfilled through the use of such enticing platforms. These bright hydrogels might provide a safer, more effective, and possibly feasible alternative for a variety of biological applications, including but not limited to early disease detection, prevention, and TE, including CTE. In addition, further research is needed before they may be used in therapeutic settings.

## Abbreviations

3D, Three-dimensional; Alg, Alginate; BASCs, Brown adipose-derived stem cells; CH, Chitosan; CMs, Cardiomyocytes; CNTs, Carbon nanotubes; CTE, Cardiac Tissue Engineering; CVDs, Cardiovascular diseases; dECM, Decellularized extracellular matrix; ECM, Extracellular matrix; EHT, Engineered heart tissue; ESCs, Embryonic stem cells; GelMA, Gelatin Methacrylate; GFs, Growth factors; Gt, Gelatin; HA, Hyaluronic acid; LV, Left Ventricular; MI, Myocardial Infarction; MSCs, Mesenchymal stem cells; PAA, Poly (acrylic acid); PAAm, Poly (acrylamide); PAni, Polyaniline; PBA-PGA, *γ*-Polyglutamic acid; PDGF-BB, Platelet-derived growth factor-BB; PEG, Polyethylene glycol.
